# Ir(III) Complexes Convert Cold to Hot Tumors via Ferroptosis/Necroptosis‐Driven Immunogenic Cell Death and Photosensitized CD47 Downregulation

**DOI:** 10.1002/advs.202514256

**Published:** 2025-11-26

**Authors:** Long‐Bo Yu, Peng Wang, Qing‐Hua Shen, Qi‐Xin Guan, Zhi‐Yuan Li, Ying‐Ying Han, Xin‐Yi Zhang, Qing‐Yuan Hu, Cai‐Ping Tan

**Affiliations:** ^1^ MOE Key Laboratory of Bioinorganic and Synthetic Chemistry School of Chemistry Sun Yat‐Sen University Guangzhou 510275 P. R. China; ^2^ Guangdong Basic Research Center of Excellence for Functional Molecular Engineering Guangzhou 510006 P. R. China; ^3^ State Key Laboratory of Anti‐Infective Drug Discovery and Development School of Pharmaceutical Sciences Sun Yat‐sen University Guangzhou 510006 P. R. China

**Keywords:** cancer immunity, CD47, hypoxia, iridium, photodynamic therapy

## Abstract

Photosensitizers engineered for spatiotemporal modulation of cluster of differentiation 47 (CD47) in hypoxic tumor microenvironments (TMEs) disrupt immunosuppressive circuits. Herein, we develop two Ir(III) complexes (**Ir1** and **Ir2**) that function as photodynamic therapy (PDT) agents via Type‐I/II mechanisms, overcoming hypoxic constraints. Under 630 nm irradiation, **Ir1**‐mediated PDT ROS‐dependently downregulates CD47, enabling spatial control that circumvents hematotoxicity. Concurrently, **Ir1**‐mediated PDT triggers immunogenic cell death (ICD) via ferroptosis‐necroptosis synergy and blocks the CD47‐SIRPα immune checkpoint, while promoting M1‐polarization of tumor‐associated macrophages. In 4T1 murine breast cancer models, **Ir1**‐PDT achieves potent tumor suppression and transforms immunologically “cold” tumors into “hot” lesions through the synergistic interplay of ICD and CD47 pathway disruption. Collectively, this work establishes a photodynamic CD47‐signaling platform for precise immune modulation, offering a clinically translatable alternative to conventional CD47‐targeting therapies.

## Graphical abstract

Red‐light‐activated **Ir1** overcomes hypoxia tolerance and adapts to the immunosuppressive tumor microenvironment, converting immunologically cold tumors into inflamed hot microenvironments. This conversion is driven by synergistic induction of immunogenic cell death through coordinated ferroptosis‐necroptosis pathways and spatiotemporally controlled downregulation of Cluster of Differentiation 47 within the tumor niche.

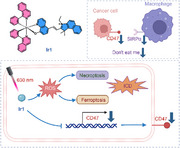

## 1 Introduction

Cluster of differentiation 47 (CD47), functioning as a “don't eat me” signal on the surface of tumor cells, binds to the signal regulatory protein alpha (SIRPα) receptor on macrophages to inhibit phagocytosis, representing a core mechanism of tumor immune evasion.^[^ ^1^, ^2^ ^]^ Targeting the CD47‐SIRPα axis is critical for reversing innate immune suppression, activating macrophage‐mediated phagocytosis and killing of tumors, and potentially synergizing with T‐cell immune responses.^[^ ^2^, ^3^, ^4^ ^]^ Current strategies include developing anti‐CD47/SIRPα monoclonal antibodies,^[^ ^5^, ^6^ ^]^ bispecific antibodies,^[^ ^7^, ^8^, ^9^ ^]^ fusion proteins,^[^ ^10^, ^11^, ^12^ ^]^ as well as combination therapies with chemotherapy,^[^ ^13^ ^]^ radiotherapy,^[^ ^3^, ^14^ ^]^ targeted drugs,^[^ ^15^, ^16^, ^17^ ^]^ or programmed death receptor 1/programmed cell death ligand 1 (PD‐1/PD‐L1) inhibitors.^[^ ^9^, ^18^, ^19^ ^]^ However, CD47 is widely expressed in normal tissues such as red blood cells, often leading to treatment‐induced severe anemia and thrombocytopenia.^[^ ^20^ ^]^ Furthermore, key challenges include M2 macrophage infiltration in solid tumors, suppression of phagocytic activity by the TMEs.^[^ ^21^, ^22^ ^]^ To address these limitations, novel approaches focus on selectively blocking CD47 at tumor sites through the design of controllably activated, microenvironment‐responsive drugs or localized delivery systems.^[^ ^23^, ^24^ ^]^ While this approach reduces off‐target toxicity to normal tissues (e.g., erythrocytes), widens the therapeutic window, and mitigates systemic immune homeostasis disruption caused by sustained CD47 inhibition,^[^ ^23^, ^24^, ^25^ ^]^ the CD47‐blocking agents released upon carrier degradation may still enter systemic circulation and cause toxic effects.

Ir(III) complexes, as highly efficient photosensitizers (PSs), generate reactive oxygen species (ROS) upon light irradiation at specific wavelengths, directly killing tumor cells and inducing immunogenic cell death (ICD).^[^ ^26^, ^27^, ^28^, ^29^, ^30^ ^]^ This process releases damage‐associated molecular patterns (DAMPs), activating dendritic cells and T cells to initiate anti‐tumor immunity.^[^ ^31^, ^32^, ^33^ ^]^ As PSs, Ir(III) complexes exhibit long‐lived excited states, high quantum yields, tunable absorption spectra (extending into the near‐infrared region), and multimodal therapeutic potential (combining photodynamic/photothermal/chemotherapeutic functions).^[^ ^29^, ^34^, ^35^, ^36^, ^37^, ^38^, ^39^ ^]^ Molecular design optimization strategies for Ir‐based photosensitizer include modulating ligand structures to red‐shift absorption wavelengths, employing nanocarriers to improve solubility and tumor accumulation and combining with immunotherapy.^[^ ^34^, ^36^ ^]^ However, Ir(III)‐based PSs also face many limitations, e.g., slow systemic clearance necessitating long‐term toxicity evaluation due to iridium's noble metal properties, reduced ROS efficiency in hypoxic TMEs, driving the need for Type I photosensitizers, and inadequate light penetration depth.^[^ ^40^, ^41^, ^42^, ^43^ ^]^

**Scheme 1:**
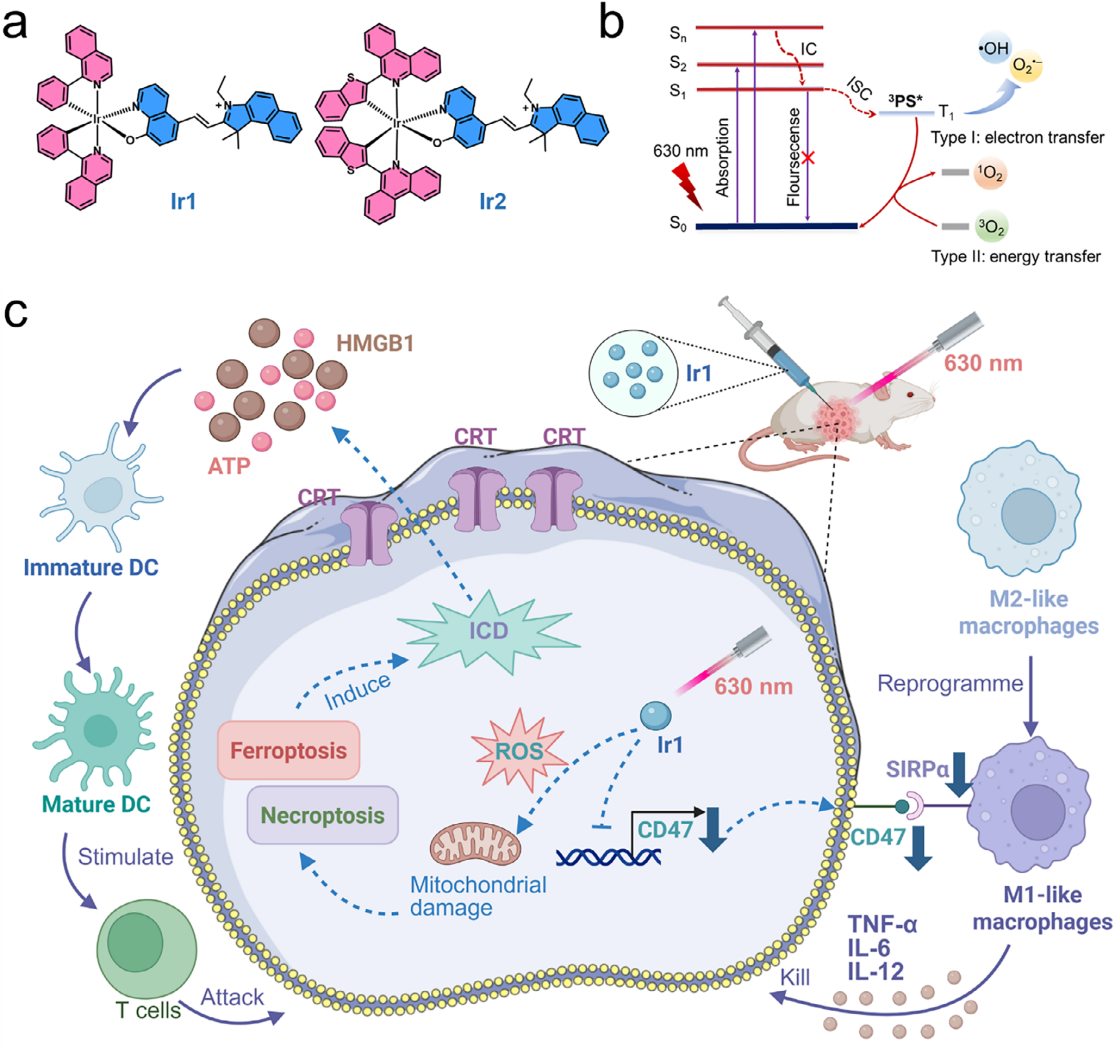
(a) Chemical structures of **Ir1** and **Ir2**. (b) The mechanism of ROS generation by **Ir1** under light irradiation is depicted in the Jablonski diagram. (c) Schematic of PDT anti‐tumor mechanism of **Ir1**. Created with BioRender.com. Abbreviation: Calreticulin (CRT); High mobility group box‐1 protein (HMGB1); Adenosine triphosphate (ATP); Interleukin‐6 (IL‐6); Interleukin‐12 (IL‐12); Tumor necrosis factor‐α (TNF‐α).

Leveraging the spatiotemporal precision of PDT, we developed photodynamically activatable CD47 inhibitors based on two red‐light‐excitable Ir(III)‐based PSs (**Ir1** and **Ir2**; Scheme **1**a). This PS‐transcription integration overcomes the hematotoxicity and systemic immune dysregulation of conventional CD47 blockers by enabling localized, transient CD47 inhibition that preserves erythrocyte integrity while potentiating solid tumor immunotherapy. Incorporation of cyanine (Cy) moieties into **Ir1** and **Ir2** red‐shifted their absorption, enabling spectral tuning and photosensitization that drive efficient Type I/II cascade reactions to overcome tumor hypoxia (Scheme 1b). Upon irradiation, **Ir1** mediates ROS‐dependent downregulation of CD47 in tumor cells. This downregulation acts in synergy with a tripartite immunostimulation: (i) ICD induction via ferroptosis‐necroptosis, (ii) CD47‐SIRPα axis blockade, and (iii) M1 repolarization of tumor‐associated macrophages (TAMs), which collectively achieve significant tumor regression and convert immunologically cold tumors into T cell‐inflamed lesions (Scheme 1c). This hypoxia‐adaptive photodynamic platform enables spatiotemporally precise immune checkpoint blockade while overcoming dose‐limiting hematological toxicities of conventional antibody therapies, offering a translatable approach for solid tumor immunotherapy.

## 2 Results and Discussion

### 2.1 **Ir1** and **Ir2** Generate Type I and II ROS upon Red‐Light Irradiation

The ligand **L** was synthesized via a condensation reaction between 3‐ethyl‐1,1,2‐trimethyl‐1H‐benzo[e]indol‐3‐ium and 8‐hydroxyquinoline‐5‐carbaldehyde under reflux conditions in ethanol, facilitated by a catalytic amount of piperidine (Scheme S1). **Ir1** and **Ir2** were obtained by reacting **L** with the corresponding precursors in a mixture solvent of CH_2_Cl_2_ and CH_3_OH. **L**, **Ir1** and **Ir2** were characterized by ESI‐MS, ^1^H/^13^C NMR and HPLC (Figures S1–S8), and elemental analysis. The UV/Vis absorption spectra of **Ir1**/**Ir2** in CH_2_Cl_2_, CH_3_OH and phosphate‐buffered saline (PBS) exhibited an intraligand (^1^IL) π→π* transition spanning 250–375 nm, and a broad metal‐to‐ligand charge transfer (^1^MLCT/^3^MLCT) band extending from 500–700 nm (Figure S9). No fluorescence emission was detected upon 630 nm excitation. Both compounds exhibited excellent stability in Dulbecco's modified eagle medium + fetal bovine serum (90% DMEM + 10% FBS) for 72 hours and maintained photostability under 630 nm irradiation for 30 minutes (Figures S10 and S11).

**Figure 1:**
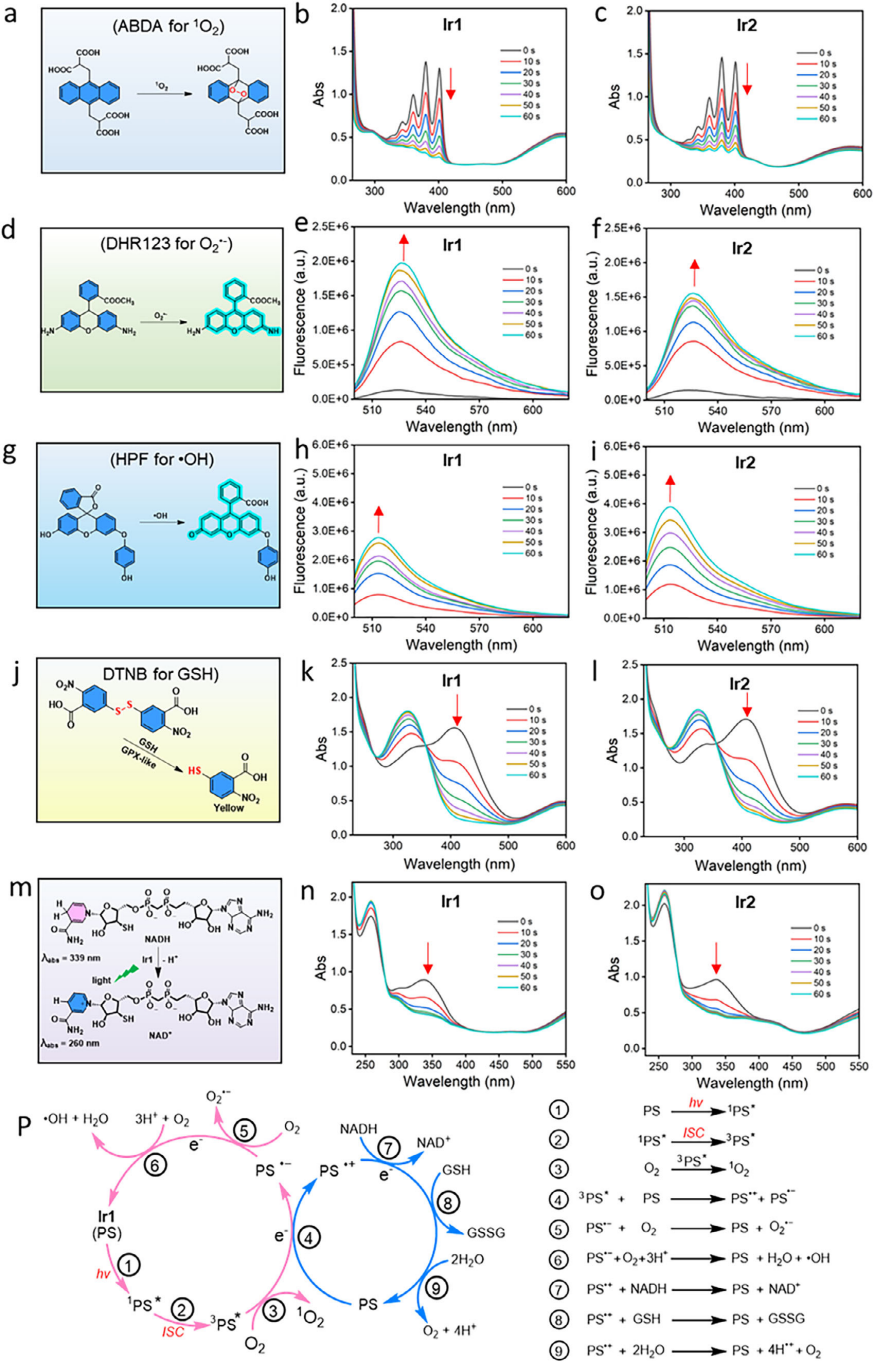
**Ir1 and Ir2 possess ROS generation and photocatalytic oxidation activity**. (a) Diagram of the reaction of ABDA with ^1^O_2_. (b‐c) The changes in the UV/vis absorption spectra of ABDA (100 µM) incubated with **Ir1** (20 µM) (b) and **Ir2** (20 µM) (c) upon light irradiation (120 mW cm^‒2^, 0–60 s) under normoxia. (d) Diagram of the reaction of DHR123 with O_2_^•−^. (e‐f) The emission spectra of DHR 123 (10 µM, λ_ex_ = 488 nm) incubated with **Ir1** (20 µM) (e) and **Ir2** (20 µM) (f) upon light irradiation (120 mW cm^−2^, 0–60 s) under normoxia. (g) Diagram of the reaction of HPF with •OH. (h‐i) The emission spectra of HPF (10 µM, λ_ex_ = 490 nm) incubated with **Ir1** (20 µM) (h) and **Ir2** (20 µM) (i) upon light irradiation (120 mW cm^−2^, 0–60 s) under normoxia. (j) Diagram of the reaction of DTNB with GSH. (k‐l) The consumption of GSH (100 µM) by **Ir1** (k) and Ir2 (l) under light irradiation (120 mW cm^−2^, 60 s) was monitored using the DTNB assay (100 µM). (m) Diagram of NADH Oxidation to NAD^+^ under Light Irradiation (120 mW cm^−2^, 60 s). (n‐o) The oxidation of NADH (100 µM) by **Ir1** (20 µM) (n) and **Ir2** (o) in PBS under light irradiation (120 mW cm^−2^, 60 s). (p) Schematic illustration of the mechanism of photoinduced ROS generation, GSH and NADH oxidation.

Upon 630 nm photoexcitation, **Ir1** and **Ir2** generated substantial amounts of singlet oxygen (^1^O_2_) under normoxic conditions within 1 min, as probed by the ^1^O_2_ indicator 9,10‐anthracenediyl‐bis(methylene)dimalonic acid (ABDA) (Figure **1**a–c). Under hypoxic conditions, the ^1^O_2_ yields of **Ir1** and **Ir2** decreased by 68.5% and 66.9%, respectively (Figure S12). **Ir1** and **Ir2** could sensitize the generation of O_2_^•‒^ and •OH upon excitation under both normoxic and hypoxic conditions, as evidenced by increased fluorescence intensities of dihydrorhodamine 123 (DHR123; the O_2_^•‒^ probe) and hydroxyphenyl fluorescein (HPF; the •OH sensor) (Figure 1d–i; Figures S13 and S14). Under the same test conditions, **L** showed no ^1^O_2_, O_2_^•‒^ and •OH generation capacity (Figures S15–S17). The capability of **Ir1** and **Ir2** to generate three types of ROS was further confirmed by electron spin resonance (ESR) analysis (Figure S18). Density functional theory (DFT) calculations also indicated that the conjugation of iridium complexes with the Cy group significantly reduced the HOMO‐LUMO energy gap, facilitating the conversion of light energy into cytotoxic ROS (Figure S19 and Table S1).

Nicotinamide adenine dinucleotide (NADH) serves as the primary electron donor in the mitochondrial electron transport chain and is an essential coenzyme for various reductases, including those involved in glycolysis.^[^ ^44^, ^45^ ^]^ The strong electron transfer activity of **Ir1** and **Ir2** prompted us to investigate their photocatalytic activity to react with NADH and glutathione (GSH), which are overexpressed in tumors. In the presence of **Ir1** and **Ir2**, light irradiation significantly oxidized NADH to NAD^+^ and GSH to glutathione disulfide (GSSG) (Figure 1j–o; Figures S20 and S21).

In all, under 630 nm irradiation, both **Ir1** and **Ir2** generate hypoxia‐tolerant triplet ROS (^1^O_2_/O_2_•^‒^/•OH) through multimodal pathways while photocatalyzing cofactor oxidation via NADH/GSH consumption (Figure 1p), demonstrating synergistic photodynamic action. Based on the mechanistic elucidation of PSs in the literature,^[^ ^46^, ^47^ ^]^ we propose that upon photoexcitation, ground‐state **Ir1** forms the singlet excited state (^1^PS*), which undergoes rapid intersystem crossing (ISC) to populate the triplet state (^3^PS*). This triplet species mediates two parallel pathways: (i) Energy transfer to molecular oxygen generates singlet oxygen (^1^O_2_); (ii) Electron transfer to adjacent ground‐state photosensitizers (PS) yields paired radical ions (PS^•+^ and PS^•‒^). The photogenerated radicals subsequently drive redox cascades: PS^•+^ oxidizes NADH to NAD^+^ and GSH to GSSG, while PS^•−^ converts O_2_ to O_2_^•−^ and •OH through electron transfer.

### 2.2 **Ir1** and **Ir2** Exhibit Potent PDT Activities under Both Normoxia and Hypoxia

The photo‐antiproliferative effects of **Ir1** and **Ir2** were quantitatively assessed using the 3‐(4,5‐dimethylthiazol‐2‐yl)‐2,5‐diphenyltetrazolium bromide (MTT) assay in triple‐negative breast cancer (TNBC) MDA‐MB‐231 (Table **1**) and 4T1 (Table S2) cells following 630 nm light irradiation. Crucially, under hypoxic conditions, **Ir1** maintained potent light‐dependent activity with nanomolar efficacy (IC_50_ < 0.05 µM) and high photocytotoxicity index (photocytotoxicity index (PI) > 265) across cell lines, significantly outperforming **Ir2**, which showed marked hypoxia sensitivity (IC_50_ > 56 µM in 4T1). **Ir1** achieved sub‐micromolar IC_50_ values under 630 nm irradiation in both normoxia (0.02 µM, MDA‐MB‐231; 0.01 µM, 4T1) and hypoxia (0.05 µM; 0.04 µM) with PI up to 997. While **Ir2** demonstrated light‐activated cytotoxicity against MDA‐MB‐231 (IC_50_ = 1.65 µM normoxia; 3.07 µM hypoxia, PI > 20), its efficacy was significantly reduced in hypoxia. Ligand **L** confirmed the iridium core's critical role through negligible phototoxicity (PI ≈ 1.3‐2.3). Activity was also assessed in human cervical cancer cells (HeLa) and mouse colon cancer cells (MC38) (Table S3 and S4). Notably, **Ir1** exhibited potent, light‐dependent anti‐proliferative activity in the nanomolar range (IC_50_ < 0.2 µM) with a high photocytotoxicity index (PI > 85.35), regardless of oxygen levels. Inductively coupled plasma‐mass spectrometry (ICP‐MS) analysis revealed 280‐fold higher cellular uptake of **Ir1** versus **Ir2** in MDA‐MB‐231 cells, respectively (Figure S22). This indicates that **Ir1**’s superior photodynamic efficacy originates from both enhanced cellular internalization and elevated ROS sensitization efficiency. The phototoxicity index of **Ir1** under red light activation (PI > 265) stands out, substantially exceeding the typical range (PI: 25–40) for this class of iridium complexes.^[^ ^48^, ^49^ ^]^

**Table 1:** (Photo)‐Antiproliferative activity (IC_50_, µM) of tested compounds against breast cancerous (MDA‐MB‐231) cell lines.

Compounds	Normoxia			Hypoxia		
	Dark^a)^	Light^b)^	PI^c)^	Dark^a)^	Light^b)^	PI^c)^
**Ir1**	11.19 ± 2.83	0.02 ± 0.003	559.50	13.25 ± 0.35	0.05 ± 0.003	265.00
**Ir2**	44.45 ± 3.80	1.65 ± 0.13	26.94	63.26 ± 8.26	3.07 ±0.07	20.61
						
**L**	13.54 ± 3.26	8.65 ± 2.86	1.57	31.36 ± 2.61	18.30 ± 1.28	1.71
Cisplatin	17.95 ± 2.17			21.15 ± 1.62		

^a)^ The cells were incubated with the compounds for 72 h without irradiation and the cell viability was detected by MTT assay;

^b)^ The cells were incubated with the compounds for 24 h in the dark and irradiated with a 630 nm laser (120 mW cm^−2^, 1 h) and then incubated for 48 h;

^c)^ PI is defined as the ratio of the IC_50_ value in the dark to that obtained in the presence of light.

### 2.3 **Ir1**‐PDT Induces Oxidative Stress and Mitochondrial Dysfunction

**Figure 2:**
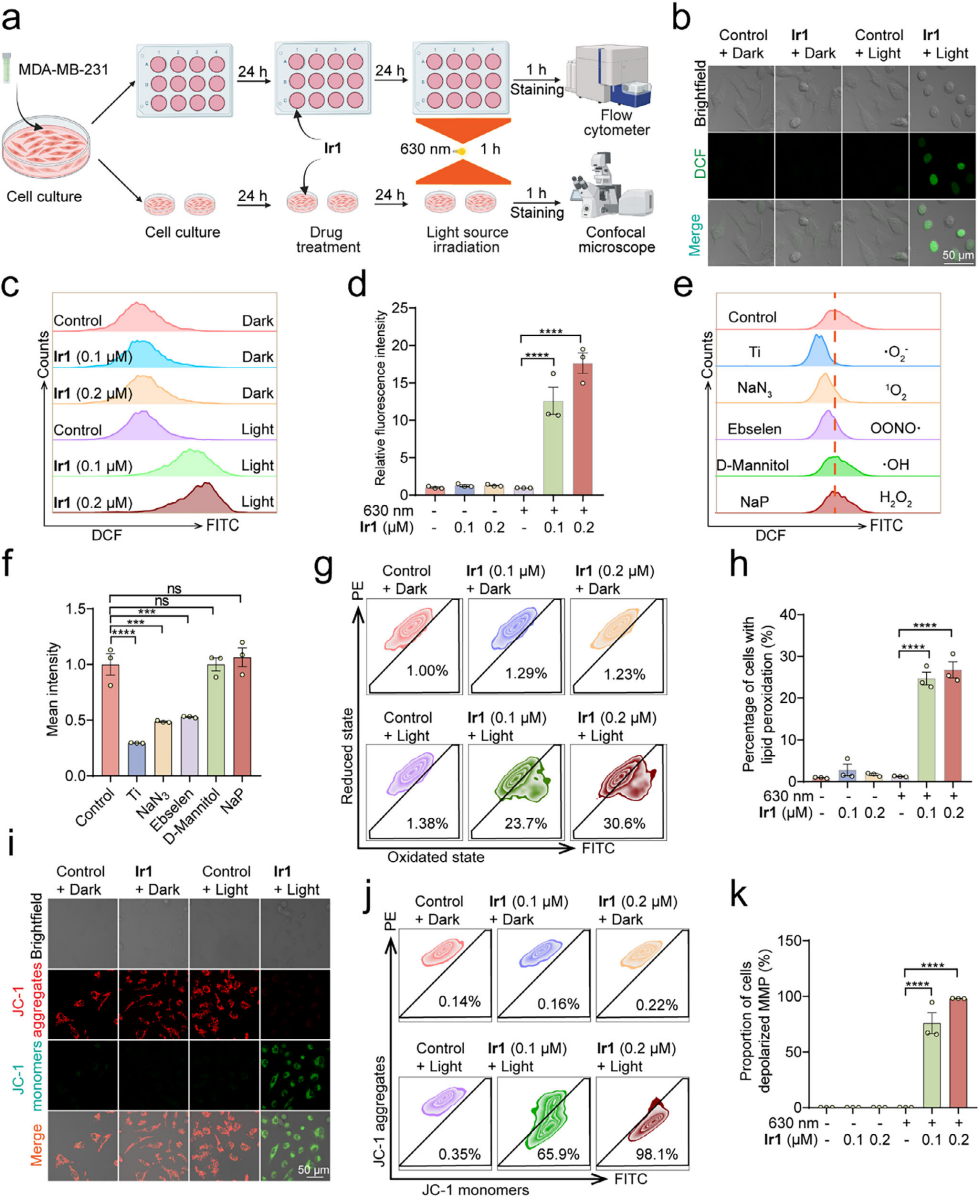
**Ir1‐PDT induces oxidative stress and mitochondrial dysfunction**. (a) Experimental workflow integrating flow cytometry and fluorescence imaging. MDA‐MB‐231 cells were cultured (24 h), treated with **Ir1** (24 h), and irradiated (630 nm, 120 mW cm^−^^2^, 1 h). After 1 h, staining was performed, followed by flow cytometry or confocal microscopy analysis (n = 3). (b‐d) Impact of **Ir1** on ROS levels measured by 2′,7′‐dichlorodihydrofluorescein diacetate (DCF, 10 µM, 30 min) staining using confocal microscopy (b) and flow cytometry (c and d). (b) DCF: λ_ex_ = 488 nm; λ_em_ = 535 ± 25 nm. Scale bar: 50 µm. **Ir1**: 0.1 µM, n = 3. (c and d) DCF: λ_ex_ = 488 nm; λ_em_ = 525 ± 40 nm, n = 3. (e and f) Effects of ROS Scavengers on **Ir1**‐induced PDT by flow cytometry. The cells were pretreated with different ROS scavengers (Tiron: 10 mM; NaN_3_: 5 mM; Ebselen: 50 µM; D‐Mannitol: 50 mM; NaP: 10 mM) for 1 h before they stained with DCFH‐DA (10 µM, 30 min). DCF: λ_ex_ = 488 nm; λ_em_ = 525 ± 40 nm. **Ir1**: 0.1 µM, n = 3 (g and h) Detection of Lipid Peroxidation in **Ir1**‐mediated PDT flow cytometry (Oxidated state: λ_ex_ = 488 nm; λ_em_ = 525 ± 40 nm. Reduced state: λ_ex_ = 561 nm; λ_em_ = 585 ± 42 nm). C11‐BODIPY 581/591 (2 µM, 30 min), n = 3. (i‐k) Impact of **Ir1**‐mediated PDT on MMP using (i) confocal microscopy (JC‐1 monomers: λ_ex_ = 514 nm; λ_em_ = 530 ± 20 nm. JC‐1 aggregates: λ_ex_ = 561 nm; λ_em_ = 590 ± 20 nm, **Ir1**: 0.1 µM) and (j and k) flow cytometry (JC‐1 monomers: λ_ex_ = 488 nm; λ_em_ = 525 ± 40 nm. JC‐1 aggregates: λ_ex_ = 561 nm; λ_em_ = 585 ± 42 nm). Scale bar: 50 µm, n = 3 Data are presented as mean ± SEM. *** *p* < 0.001, **** *p* < 0.0001.

Considering the higher PDT effects of **Ir1**, we systematically evaluated its pharmacological properties following the experimental protocol (Figure **2**a). Confocal fluorescence microscopy revealed substantial intracellular ROS generation in **Ir1**‐PDT‐treated cells (Figure 2b). Quantitative flow cytometry demonstrated that **Ir1**‐PDT induced dose‐dependent ROS production under normoxia, showing a 17.6‐fold increase (0.2 µM) versus dark controls (Figure 2c,d). ROS scavenger efficacy followed the hierarchy: Tiron (superoxide anion/O_2_•^−^) > NaN_3_ (singlet oxygen/^1^O_2_) > Ebselen (peroxynitrite/ONOO^−^), confirming **Ir1**‐PDT primarily generates these three ROS species (Figure 2e,f). Furthermore, **Ir1**‐PDT triggered concentration‐dependent lipid peroxidation, with 26.8% of cells showing lipid peroxide positivity at 0.2 µM (Figure 2g,h). Critically, ROS production remained robust under hypoxic conditions (Figure S23), confirming oxygen‐independent activation of oxidative stress pathways.

Confocal microscopy imaging confirmed dose‐dependent damage to mitochondria caused by **Ir1‐**PDT treatment, which was quantitatively supported by flow cytometric analysis of mitochondrial membrane potential (MMP) loss under normal conditions (Figure 2i–k) and hypoxic conditions (Figure S24). Notably, MMP collapse was observed in 98.1% of cells at 0.2 µM **Ir1** under PDT conditions under normal conditions (Figure 2k).

### 2.4 **Ir1**‐Mediated PDT Induces Ferroptosis and Necroptosis to Initiate ICD

**Figure 3:**
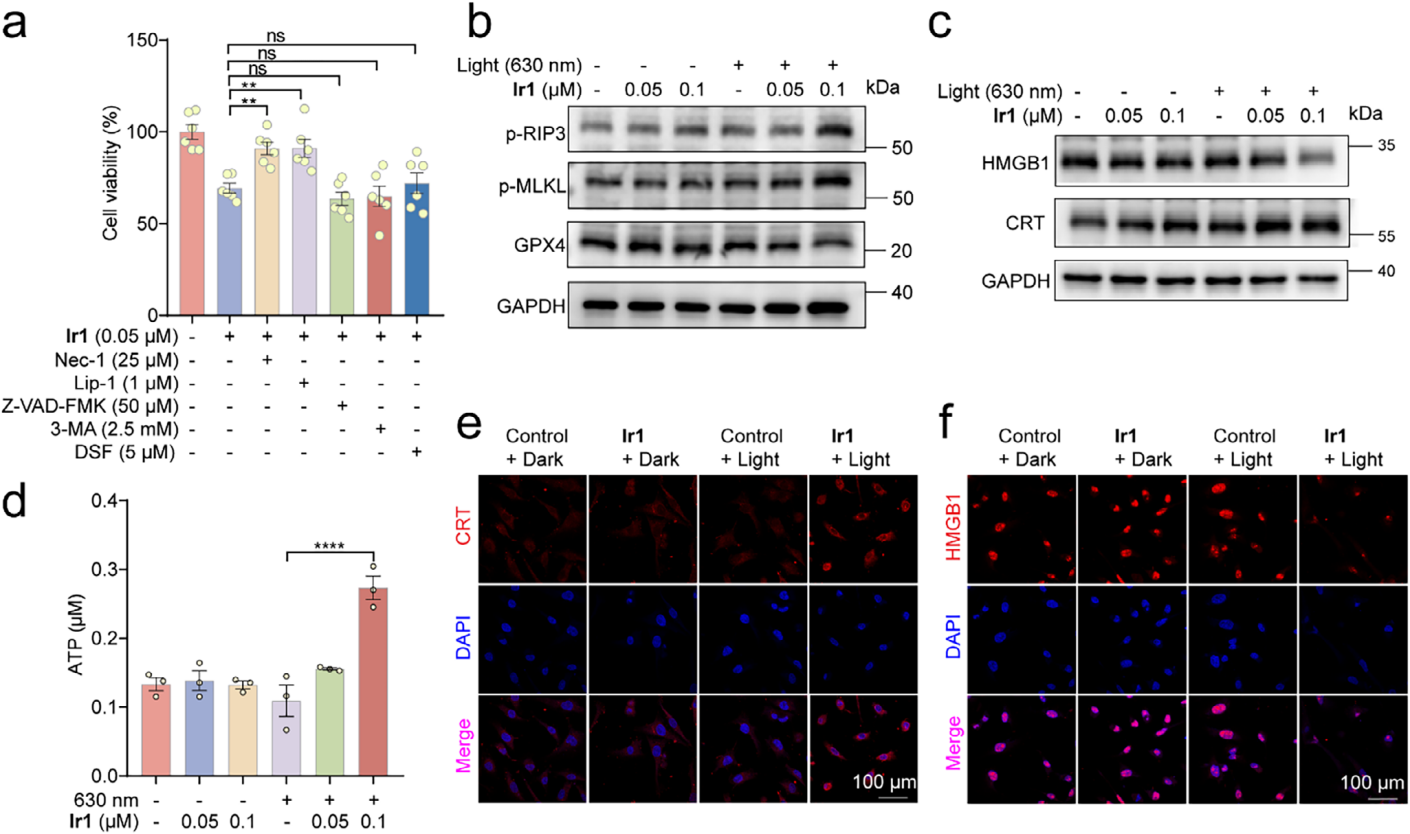
**Ir1‐mediated PDT induces ferroptosis and necroptosis to initiate ICD in MDA‐MB‐231 cells**. (a) Effects of pharmacological inhibitors on the antiproliferative efficacy of **Ir1**‐PDT (n = 6). (b and c) Western blot analysis of protein expression profiles modulated by **Ir1**‐PDT (n = 3). (d) Extracellular ATP levels in MDA‐MB‐231 cell supernatants (n = 3). (e and f) Immunofluorescence analysis of CRT (e) and HMGB1 (f) expression following **Ir1**‐PDT treatment. **Ir1**: 0.1 µM, n = 3. Scale bar: 100 µm. Data are presented as mean ± SEM. ** *p* < 0.01, **** *p* < 0.0001. CRT/HMGB1: λ_ex_ = 561 nm; λ_em_ = 585 ± 42 nm.

To delineate the cell death mechanism triggered by **Ir1** under PDT conditions, cell death‐specific blockers were employed, including inhibitors for necroptosis (Necrostatin‐1; Nec‐1), ferroptosis (Liproxstatin‐1; Lip‐1), apoptosis (Z‐VAD‐FMK), autophagy (3‐Methyladenine; 3‐MA) and pyroptosis (Disulfiram; DSF). Both necroptosis inhibitor Necrostatin‐1 (Nec‐1) and ferroptosis inhibitor Liproxstatin‐1 (Lip‐1) significantly mitigated **Ir1**‐mediated photocytotoxicity (Figure **3**a). Western blotting analysis further revealed coordinated activation of dual death pathways by **Ir1**‐mediated PDT, evidenced by elevated phosphorylation of necroptosis markers (RIP3 and MLKL) coupled with reduced GPX4 expression—a key ferroptosis regulator (Figure 3b). Both ferroptosis and necroptosis can be immunogenic forms of cell death, releasing DAMPs that trigger inflammatory and immune responses.^[^ ^50^ ^]^ Immunoblotting and confocal microscopy analyses confirmed the decrease of HMGB1 in nucleus and substantial cell surface exposure of CRT following **Ir1**‐PDT treatment (Figure 3c,e,f). Importantly, we observed a 2.1‐fold increase in extracellular ATP secretion in the culture supernatant of **Ir1**‐treated cells, further supporting robust ICD induction (Figure 3d).

### 2.5 RNA‐Sequencing

**Figure 4:**
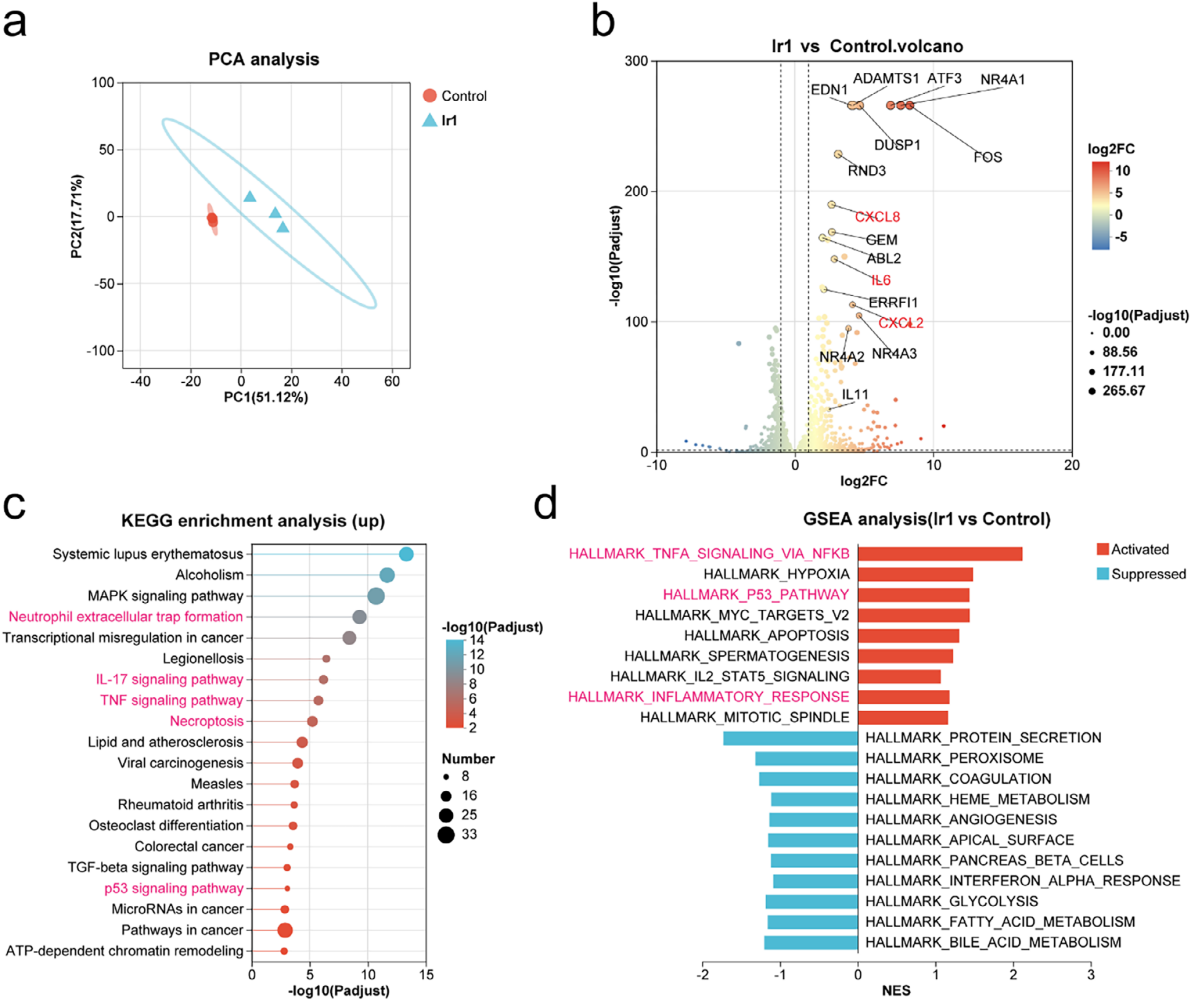
Transcriptomic impact of **Ir1**‐mediated PDT. MDA‐MB‐231 cells were cultured (24 h), treated with **Ir1** (0.1 µM, 24 h), and irradiated (630 nm, 120 mW cm^−^^2^, 1 h). After 1 h, samples were collected for RNA sequencing (n = 3)**. (a)** Inter‐sample correlation analysis. **(b)** Volcano plot displaying significantly differentially expressed genes (DEGs, |log_2_FC| > 1, adjusted *p* < 0.05) from RNA‐seq analysis. **(c)** KEGG pathway enrichment of up‐regulated genes. Pathways are ordered by increasing adjusted *p*‐value (top 20 shown). **(d)** GSEA using hallmark gene sets. Pathways are ranked by significance (top 20 enriched pathways shown by adjusted *p*‐value).

RNA sequencing (RNA‐seq) was employed to characterize the transcriptomic impact of **Ir1** under PDT conditions. Principal component analysis (PCA) revealed distinct segregation between treatment and control groups, indicating robust data reproducibility (Figure **4**a). Differential gene expression analysis revealed significant upregulation of key pro‐inflammatory cytokines, including C‐X‐C motif chemokine ligand 8 (CXCL8), IL‐6, and C‐X‐C motif chemokine ligand 2 (CXCL2) (|log_2_ fold change (FC)| > 1, *p* < 0.05; Figure 4b), suggesting a robust immune‐stimulatory effect. Kyoto Encyclopedia of Genes and Genomes (KEGG) enrichment analysis further indicated that **Ir1**‐PDT significantly enhanced neutrophil extracellular trap (NET) formation and activated immune‐related pathways such as the interleukin‐17 (IL‐17) and tumor necrosis factor (TNF) signaling pathways (Figure 4c). Additionally, we observed upregulation of necroptosis, consistent with our earlier findings, and downregulation of the p53 signaling pathway, which may contribute to proliferation inhibition. Gene Set Enrichment Analysis (GSEA) corroborated these results, confirming that TNF‐α signaling promotes nuclear factor kappaB (NF‐κB) ‐mediated inflammatory responses and modulates the p53 pathway (Figure 4d).

### 2.6 **Ir1**‐PDT Downregulates CD47 Expression and Promotes M1 Macrophage Polarization

**Figure 5:**
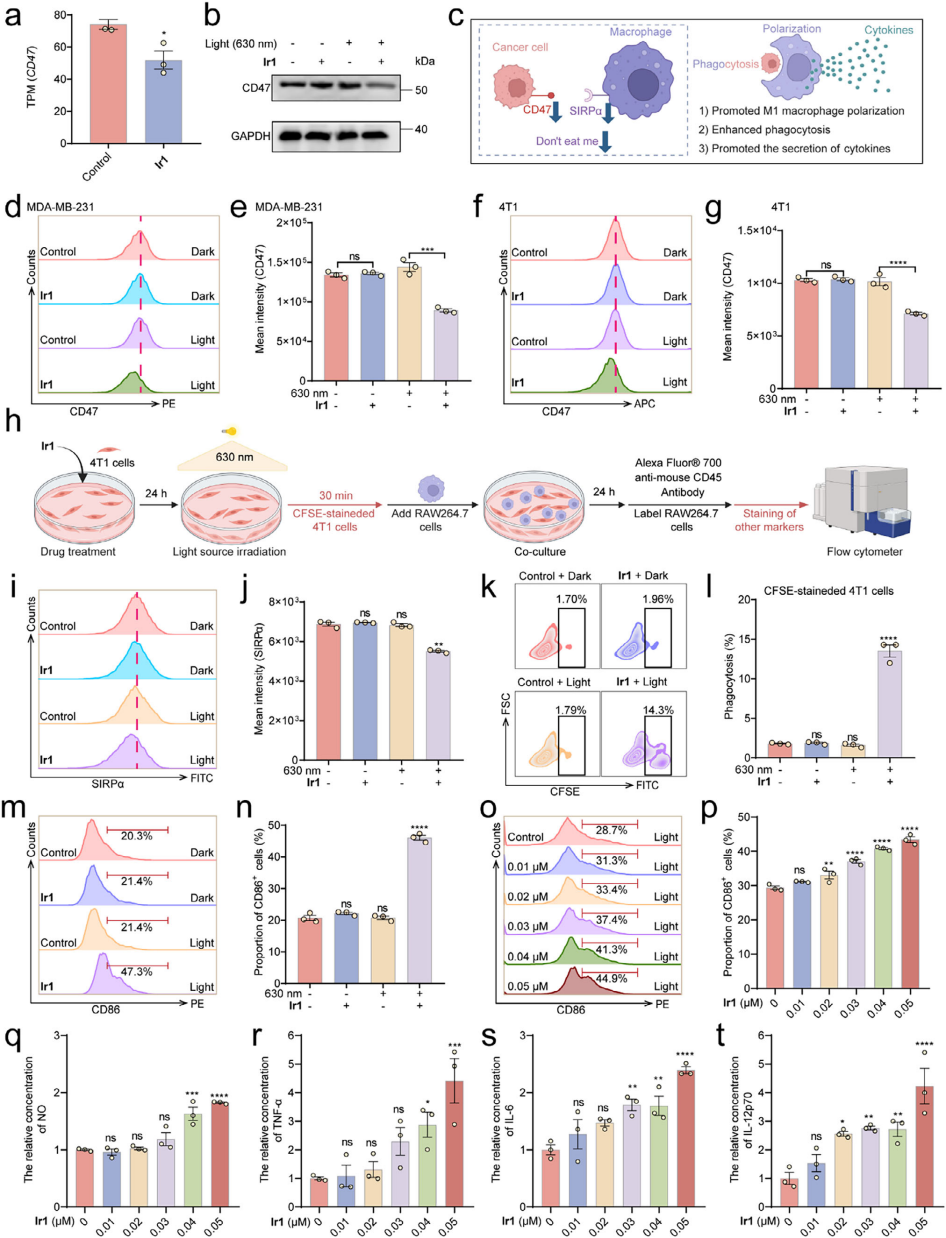
**Ir1‐PDT downregulates CD47 expression and promotes M1 macrophage polarization**. (a) CD47 gene expression level (transcripts per million, TPM, n = 3). (b) Representative Western blot images of CD47. **Ir1**: 0.1 µM, n = 3. (c) CD47‐SIRPα “Don't Eat Me” Signaling. (d and f) Flow cytometric analysis of CD47 expression. **Ir1**: 0.1 µM, n = 3. (e and g) Quantification of CD47 mean fluorescence intensity (n = 3). (h) Experimental flowchart (i‐t). 4T1 cells were cultured (24 h), treated with **Ir1** (0.05 µM, 24 h), and irradiated (630 nm, 120 mW cm^−^^2^, 1 h). RAW264.7 cells were co‐cultured with 4T1 cells and treated for 24 hours prior to sample collection and staining. The flow cytometry dot plot clearly demarcated the differential distribution profiles of RAW264.7 cells (CD45^+^) and 4T1 cells (CD45^−^) in their co‐culture system (n = 3). (i) Flow cytometric analysis of SIRPα expression (n = 3). (j) Quantification of SIRPα mean fluorescence intensity (n = 3). (k) Flow cytometric analysis of phagocytic activity in RAW264.7 cells (n = 3). (l) Statistical analysis of phagocytic activity in RAW264.7 cells (n = 3). (m‐p) Statistical analysis of M1 macrophages (n = 3). (q‐t) Detection of cytokines in cell supernatant (n = 3). Data are presented as mean ± SEM. * *p* < 0.05, ** *p* < 0.01, *** *p* < 0.001, **** *p* < 0.0001.

RNA sequencing analysis revealed that **Ir1**‐PDT significantly downregulated *CD47* expression (0.70‐fold), a notable finding given that CD47 is frequently overexpressed in breast tumors and associated with immune evasion and poor prognosis (Figure **5**a). KEGG enrichment analysis demonstrated that **Ir1**‐PDT significantly suppressed the ECM‐receptor interaction pathway (*p* < 0.05, top 2 shown), a key pathway involved in CD47‐dependent cell adhesion and immune evasion (Figure S25). Western blot and flow cytometry confirmed this suppression at the protein level, with CD47 surface expression reduced to 0.67‐fold in MDA‐MB‐231 cells and 0.71‐fold in 4T1 cells following **Ir1**‐PDT treatment (Figure 5b–g). Conversely, the CD47 downregulation induced by **Ir1**‐PDT was effectively reversed by Tiron, a scavenger of superoxide anions, thereby definitively establishing the process as ROS‐dependent (Figure S26) CD47 is widely expressed in normal tissues, including red blood cells, and its systemic blockade often leads to severe anemia and thrombocytopenia.^[^ ^20^ ^]^ To evaluate potential hematological toxicity of the PS, we intravenously administered **Ir1** (3 mg kg^−1^) to mice via tail vein injection and analyzed red blood cell and platelet 48 hours post‐treatment. Notably, **Ir1** at this dosage showed no significant effects on platelet or erythrocyte levels, demonstrating its superior safety profile compared to conventional CD47‐targeted therapies (Figure S27).

To investigate the effect of tumor cells on macrophages, we established a co‐culture system of 4T1 cells and RAW264.7 cells, using CD45 as a marker (CD45‐positive cells were RAW264.7 cells, while CD45‐negative cells were 4T1 cells; Figure 5h). Since CD47‐SIRPα interaction inhibits macrophage phagocytosis by transmitting a “don't‐eat‐me” signal, its downregulation weakened this immunosuppressive axis, as evidenced by a decrease in SIRPα fluorescence intensity (0.81‐fold) in RAW264.7 macrophages co‐cultured with treated 4T1 cells (Figure 5c,i–j).

This disruption of immune checkpoint signaling enhanced macrophage‐mediated tumor clearance, with phagocytosis of **Ir1‐**PDT‐treated 4T1 cells increasing by 7.5‐fold (Figure 5c,k,l,). Moreover, macrophages exhibited M1 polarization, marked by a 2.2‐fold upregulation of CD86, a trend that intensified with higher **Ir1** concentrations (Figure 5c,m–p). Additionally, quantitative analysis revealed that **Ir1**‐PDT triggered dose‐dependent increases in NO levels (1.8‐fold at 0.05 µM), paralleled by substantial elevation of pro‐inflammatory mediators including TNF‐α (4.4‐fold), IL‐12p70 (4.2‐fold), and IL‐6 (2.4‐fold) (Figure 5c,q–t). These findings collectively indicate that **Ir1‐**PDT remodels the tumor immune microenvironment by suppressing CD47, thereby enhancing macrophage phagocytosis, promoting M1 polarization, and stimulating pro‐inflammatory cytokine release—a multi‐faceted mechanism that could improve therapeutic outcomes in breast cancer (Figure 5c).

### 2.7 **Ir1**‐PDT Exerts Potent in Vivo Antitumor and Immunostimulatory Effects

**Figure 6:**
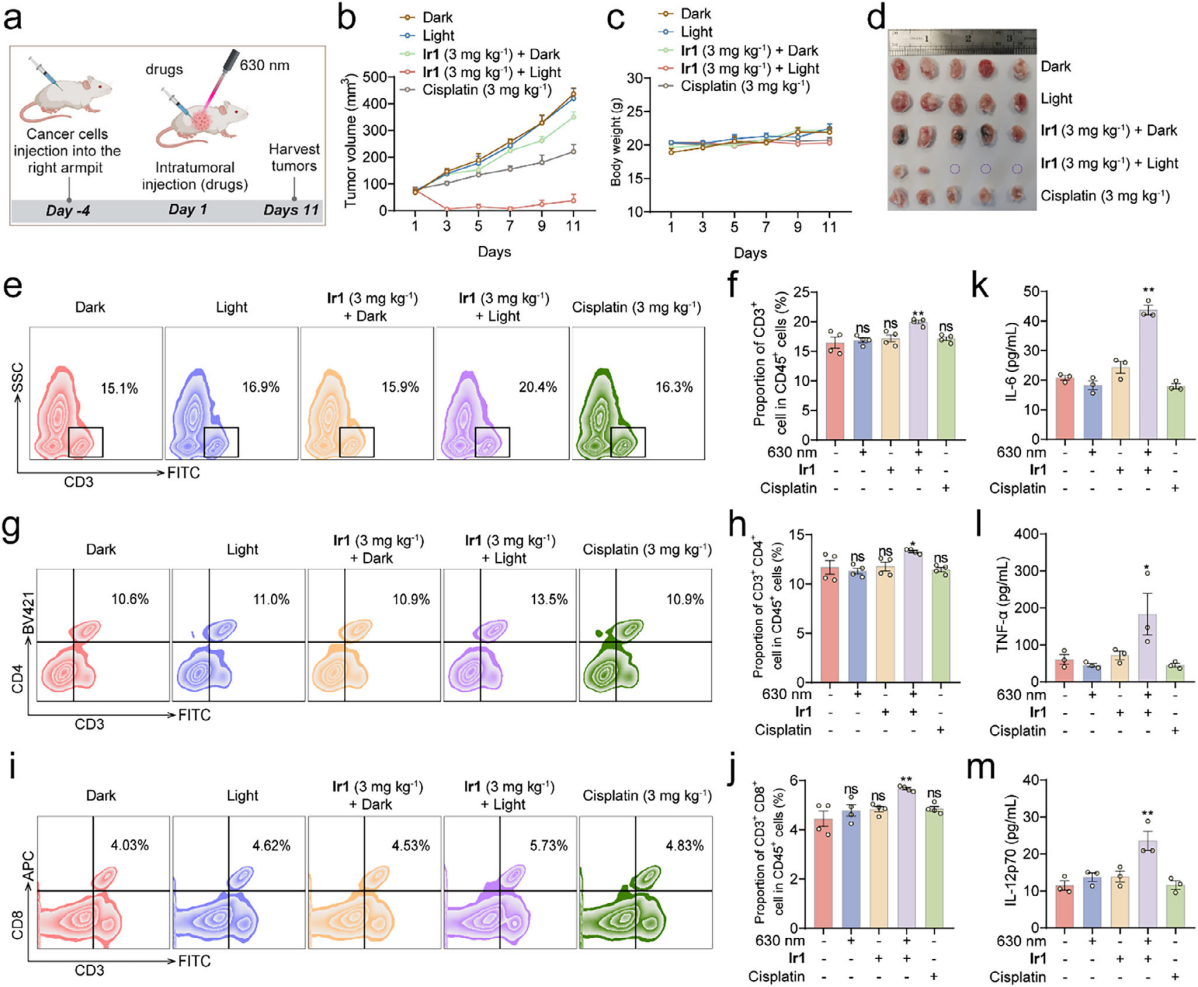
***In vivo* antitumor and immunostimulatory effects of Ir1‐PDT**. (a) Schedule of in vivo evaluation. (b) Tumor growth curves over time in different treatment groups (n = 5). (c) Body weight changes in mice during the experimental period (n = 5). (d) Photographic documentation of tumors harvested at endpoint (Day 11, n = 5). (e‐j) Proportion of immune cell subsets within CD45⁺ cells in spleens (n = 4). (k‐m) Quantification of serum cytokine levels by ELISA (n = 3). Groups: Dark (i), Light (ii), **Ir1** (3 mg kg^−1^) + dark (iii), **Ir1** (3 mg kg^−1^) + light (iv) and cisplatin (3 mg kg^−1^) (v). Data are presented as mean ± SEM. * *p* < 0.05, ** *p* < 0.01.

In vivo antitumor efficacy was evaluated using a unilateral transplanted model. Potent therapeutic effects were observed following a single administration of **Ir1** and photo‐irradiation on day 1. Terminal analysis at day 11 included flow cytometry of blood and spleen specimens to quantify acquired immune activation (Figure **6**a). **Ir1**‐PDT demonstrated potent antitumor efficacy, achieving a remarkable 91.4% tumor inhibition rate, with complete tumor regression observed in 60% (3/5) of mice (Figure 6b,d). In contrast, the dark‐treated **Ir1** group exhibited only about 20.0% inhibition rate, while cisplatin showed moderate activity (49.3%). Light treatment alone (control group) had no significant effect on tumor growth. No significant body weight fluctuations or organ toxicity were observed in all the groups, as confirmed by H&E staining of major tissues (heart, liver, spleen, lungs, and kidneys; Figure 6c; Figure S28).

Beyond direct tumor suppression, **Ir1**‐PDT robustly activated systemic antitumor immunity (Figure 6e–m). Flow cytometry analysis of spleen revealed increased proportions of CD3^+^ T cells, CD4^+^ helper T cells, and CD8^+^ cytotoxic T cells, indicating enhanced adaptive immune responses (Figure 6e–j). Furthermore, serum levels of key pro‐inflammatory cytokines were significantly elevated—TNF‐α (3.0‐fold), IL‐6 (2.1‐fold), and IL‐12p70 (2.1‐fold)—highlighting the induction of a potent immunostimulatory milieu (Figure 6k–m). In all, **Ir1**‐PDT treatment potently eradicates tumors and transforms immunologically “cold” tumors into “hot” tumors through systemic T‐cell recruitment and cytokine activation, enabling immune‐mediated control without toxicity.

In a model of established tumors (about 400 mm^3^), **Ir1**‐PDT exhibited potent antitumor efficacy, inhibiting tumor growth by 68.4% without causing significant body weight loss (Figure S29a–d). Histological and immunohistochemical analyses demonstrated extensive tumor damage, which was associated with the induction of ferroptosis and necroptosis (Figure S29e,f). The treatment also proved to be safe, with no significant toxicity detected in major organs (Figure S29g). These findings establish that **Ir1** retains its efficacy against larger, established tumors.

### 2.8 **Ir1**‐PDT Establishes Protective Immunity Against Tumor Rechallenge

**Figure 7:**
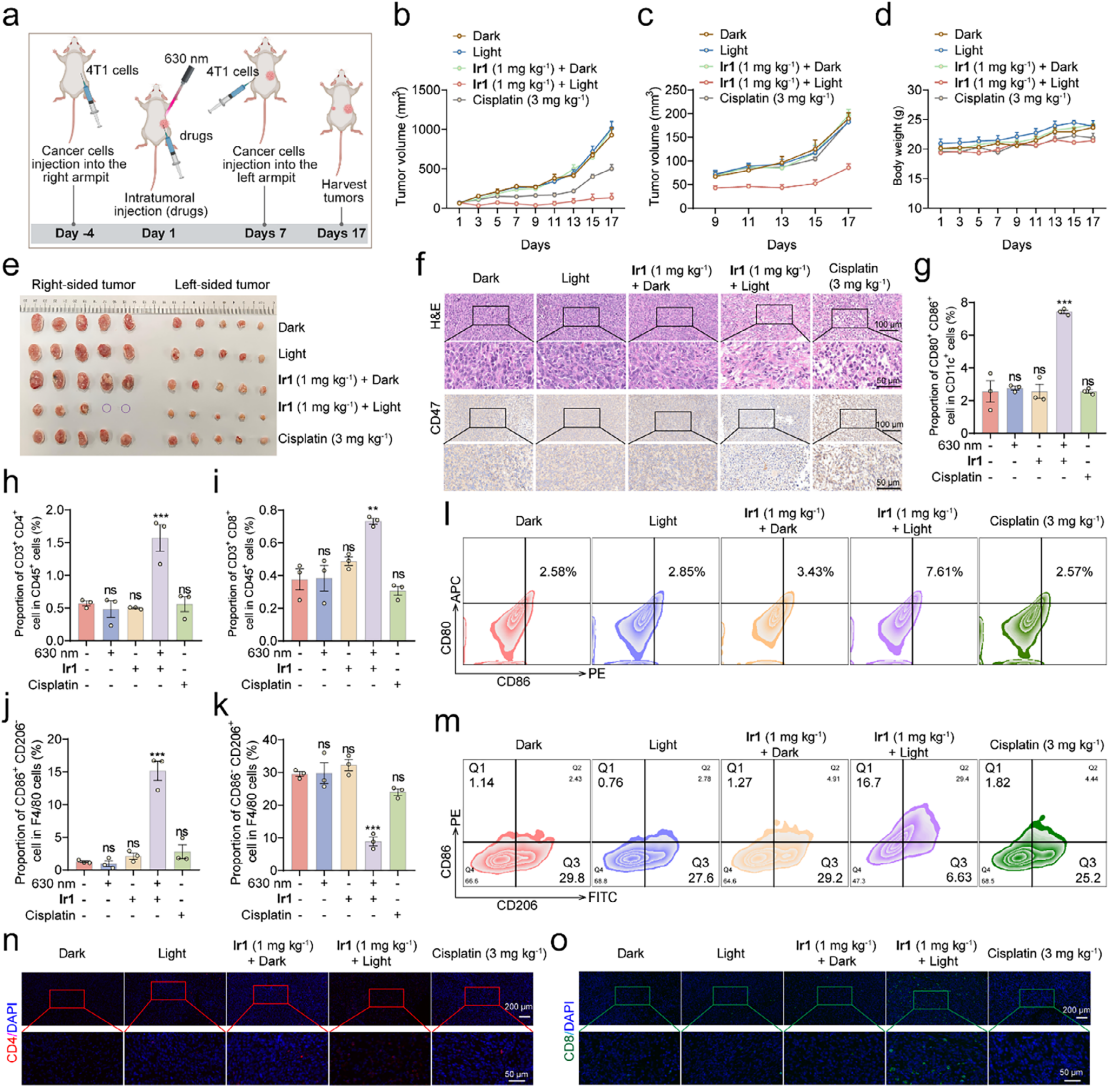
**Ir1‐PDT establishes protective immunity against tumor rechallenge**. (a) Schedule of in vivo evaluation. (b) Right‐sided tumor growth curves over time in different treatment groups (n = 5). (c) Left‐sided tumor growth curves over time in different treatment groups (n = 5). (d) Body weight changes in mice during the experimental period (n = 5). (e) Photographic documentation of tumors harvested at endpoint (Day 17, n = 5). (f) Immunohistochemical and hematoxylin and eosin (H&E) staining of right‐sided tumors. Scale bar: 50 µm. (g‐m) Proportion of immune cell subsets in right‐sided tumors. (n and o) Representative immunofluorescence images of left‐sided tumor‐infiltrating (n) CD4^+^ (red) and (o) CD8^+^ (green) T cells. Nuclei counterstained with 4′,6‐diamidino‐2‐phenylindole (DAPI, blue). Scale bar: 50 µm. Groups: Dark (i), Light (ii), **Ir1** (1 mg kg^−1^) + dark (iii), **Ir1** (1 mg kg^−1^) + light (iv) and cisplatin (3 mg kg^−1^) (v). Data are presented as mean ± SEM. ** *p* < 0.01, *** *p* < 0.001.

To further assess its acquired immune effects, we established a tumor rechallenge model to evaluate protective immunity (Figure **7**a). On day ‐4, 4T1 cells were implanted into the right axilla of mice, followed by a single intratumoral treatment on day 1 with either **Ir1** (1 mg kg^−1^) combined with light irradiation or appropriate controls for treatment. A secondary challenge was performed on day 7 by implanting 4T1 cells into the left axilla. Body weight and tumor volume were monitored until sample collection on day 17. In right‐sided tumors, **Ir1‐**PDT achieved an 85.7% tumor inhibition rate, with complete regression observed in two of five mice (Figure 7b,e). In contrast, cisplatin treatment resulted in a 46.1% inhibition rate. Neither the **Ir1** dark control nor light‐only group exhibited significant suppression (Figure 7b,e). Notably, left‐sided tumors in the **Ir1**‐PDT group showed a 54.9% inhibition rate, confirming its ability to induce systemic antitumor immunity (Figure 7c,e). No significant weight loss was observed, supporting the treatment's safety (Figure 7d). Histopathological analysis via H&E staining revealed substantial tumor tissue damage in both **Ir1**‐PDT and cisplatin groups (Figure 7f). Immunohistochemistry further demonstrated a significant reduction in CD47 expression following **Ir1**‐PDT, whereas other groups remained unchanged (Figure 7f). Furthermore, immunohistochemistry revealed that the **Ir1**‐PDT decreased GPX4 and increased p‐MLKL levels in tumor tissues, consistent with the promotion of both ferroptosis and necroptosis (Figure S30).

Flow cytometry analysis revealed that **Ir1**‐PDT enhanced dendritic cell (DC) maturation by 2.8‐fold compared to controls, with no notable effects in other treatment groups (Figure 7g,l). As key antigen‐presenting cells, these activated DCs potentiated T cell‐mediated antitumor responses, evidenced by a 3.1‐fold increase in CD3^+^ CD4^+^ T cells and a 1.9‐fold rise in CD3^+^ CD8^+^ T cells in **Ir1**‐PDT group (Figure 7h,i; Figure S31). Furthermore, **Ir1**‐PDT markedly altered macrophage polarization within tumor‐associated macrophages (F4/80^+^), inducing a 12.0‐fold upregulation of M1‐type (CD86^+^ CD206^−^) macrophages while suppressing M2‐type (CD86^−^ CD206^+^) populations to 30% of control levels (Figure 7j,k,m,). Critically, immunofluorescence analysis of left‐sided tumors confirmed enhanced infiltration of CD4^+^ and CD8^+^ T cells in the **Ir1‐**PDT group, reinforcing its role in promoting systemic antitumor immunity (Figure 7n,o). In all, **Ir1**‐PDT induces robust systemic antitumor immunity, eradicating primary tumors and suppressing distant growth while reprogramming the tumor immune microenvironment.

## 3 Conclusion 

We engineered two hypoxia‐tolerant Ir(III) PSs (**Ir1**/**Ir2**) enabling spatiotemporal modulation of CD47. Through red light activation, **Ir1** disrupts the “CD47‐SIRPα” immunosuppressive axis, which synergizes with ferroptosis/necroptosis‐driven ICD to collectively drive cold‐to‐hot tumor conversion. This PDT strategy overcomes hypoxia via sequential Type I/II ROS, while concurrently inducing localized CD47 downregulation (thereby preserving systemic CD47 on erythrocytes) and triggering ICD. Mechanistically, photoactivated **Ir1** blocks CD47‐SIRPα checkpoint signaling and promotes M1 macrophage polarization, thereby enhancing innate immune activation alongside ICD‐driven adaptive immunity. In vivo studies demonstrated potent tumor suppression and transformation of cold tumors into immunogenic hot microenvironments characterized by robust T cell infiltration and cytokine release. Our work establishes a light‐controlled precision immunotherapy paradigm as a viable clinical alternative to conventional CD47‐targeting approaches.

## 4 Experimental Section/Methods

### Materials

IrCl_3_·3H_2_O, 1,1,2‐trimethyl‐1H‐benzo[e]indole, iodoethane, 9,10‐anthracenedipropanoic acid (ABDA), and 8‐hydroxyquinoline‐5‐carbaldehyde were purchased from Aladdin Chemical (China). 1‐Benzothiophen‐2‐ylboronic acid, 1‐Phenylisoquinoline, dihydrorhodamine 123 (DHR123), and 6‐chlorophenanthridine were purchased from Macklin (China). 1‐Phenylisoquinoline and tetrakis(triphenylphosphine)palladium were obtained from BIDE (China). Dimethylsulfoxide‐*d*_6_ (DMSO‐*d*_6_) was supplied by Sigma‐Aldrich (USA). 5,5‐Dimethyl‐1‐pyrroline N‐oxide (DMPO, 97%) was purchased from J&K Scientific (China). Hydrogen peroxide (30%), hydrochloric acid (36%), nitric acid (68%), sulfuric acid (98%), methanol, dichloromethane, 2‐ethoxyethanol, ethanol, acetonitrile, and toluene were purchased from Guangzhou Chemical Reagent Factory (China). The antibodies and reagents used in this study were obtained from multiple commercial sources. From BioLegend (San Diego, CA), we acquired TruStain FcX™ (anti‐mouse CD16/32, 101 319), APC anti‐mouse CD47 (127 514), APC anti‐mouse CD80 (104 714), PE anti‐mouse CD86 (105 008), FITC anti‐mouse CD3 (100 204), BV421 anti‐mouse CD4 (562 891), PE anti‐mouse CD4 (100 408), FITC anti‐mouse CD206 (141 703), APC anti‐mouse CD8a (162 306), Alexa Fluor® 700 anti‐mouse CD45 (103 128), and FITC anti‐mouse CD11c (117 305), while FITC anti‐SIRPα/SHPS‐1 was purchased from Sino Biological (Beijing, China) and PE anti‐human CD47 (E‐AB‐F1413D) from Elabscience (Wuhan, China). Additional key antibodies included phospho‐MLKL (Ser358, AF7420), phospho‐RIPK3 (AF7443), and GAPDH (AF7021) from Affinity Biosciences (Cincinnati, OH); CD47 (20305‐1‐AP) and GPX4 (67763‐1‐Ig) from Proteintech (Wuhan, China); and calreticulin (CRT) and HMGB1 from Abcam (Cambridge, UK). Secondary antibodies Cy3‐labeled goat anti‐rabbit IgG (GB21303) and HRP‐conjugated rabbit anti‐goat IgG (GB23204) along with SDS‐PAGE transfer buffer powder (G2017‐1L), running buffer (G2018‐15), electron microscope fixative (G1102‐100 mL), and PBS powder (G0002) were obtained from Servicebio (Wuhan, China). Various reagents including MTT (ST1537), goat serum (C0265), QuickBloc^TM^ blocking solution (P0260), RIPA lysis buffer (P0013B), SDS‐PAGE gel preparation kit (P0012A), antifade mounting medium (P0126‐5 mL), DAPI (C1002), BCA protein assay kit (P0010), JC‐1 mitochondrial membrane potential assay kit (C2006), ROS assay kit (DCFH‐DA), and NO detection kit were purchased from Beyotime Biotechnology (Shanghai, China). Other essential reagents included BSA (B24726) from ABCONE (Shanghai, China), 4% paraformaldehyde (BL539A) from Biosharp (Hefei, China), Z‐VAD(OMe)‐FMK (HY‐16658) from MedChemExpress (NJ, USA), cisplatin (D807330) from Macklin (Shanghai, China), necrostatin‐1 (S8037) from Selleck (Houston, TX), liproxstatin‐1 (B4987) and 3‐methyladenine (A8353) from APExBIO (Houston, TX), D‐mannitol and Tiron from J&K Co., Ltd (Beijing, China), ebselen from MedChemExpress (NJ, USA), and sodium pyruvate (NaP) and D‐mannitol from Sigma‐Aldrich (St. Louis, MO). The mouse IL‐6, IL‐12/p70 and TNF‐α ELISA kit was purchased from Jiangsu Meimian Industrial Co., Ltd (Yanchen, China), while CFSE (abs9106) was obtained from Absin (Shanghai, China). All reagents were selected based on their established performance in previous studies and compatibility with our experimental protocols.

### General Instruments

The quoted m/z values represented the major peaks in the isotopic distribution. Nuclear magnetic resonance (NMR) spectra were recorded on a Bruker Avance III 400 MHz spectrometer (Germany). Shifts were referenced relative to the internal solvent signals. Electrospray ionization mass spectrometry (ESI‐MS) was recorded on a Thermo Scientific LTQ linear ion trap mass spectrometer (USA). UV/Vis absorption spectra were obtained on a UV‐2600 spectrophotometer (Shimadzu, Japan). The electron spin resonance (ESR) spectra were obtained on an electron paramagnetic resonance spectrometer (JES‐FA200, JEOL, Japan).

### Synthetic Procedures

The general synthetic route of **L**, **Ir1** and **Ir2** were shown in Scheme S1. The ligands **L** was prepared according to previous papers with slight modifications.^[^ ^51^ ^]^ **(E)‐3‐ethyl‐2‐(2‐(8‐hydroxyquinolin‐5‐yl)vinyl)‐1,1‐dimethyl‐1H‐benzo[e]indol‐3‐ium (L**): ^1^H NMR (400 MHz, Chloroform‐d) δ 9.11 (s, 1H), 8.49 (d, J = 13.9 Hz, 1H), 8.38 (d, J = 8.9 Hz, 1H), 8.22 (d, J = 10.3 Hz, 1H), 8.11 (d, J = 8.9 Hz, 1H), 7.92 (d, J = 8.7 Hz, 2H), 7.61 (d, J = 6.9 Hz, 2H), 7.44 (t, J = 7.7 Hz, 1H), 7.30 (d, J = 8.8 Hz, 1H), 6.81 (d, J = 9.8 Hz, 1H), 6.46 (d, J = 13.5 Hz, 1H), 4.16 (q, J = 6.9 Hz, 2H), 2.07 (s, 6H), 1.51 (t, J = 7.5 Hz, 3H). **6‐(benzo[b]thiophen‐2‐yl)phenanthridine (btp)**: **btp** was prepared with minor adjustments based on methods reported in the literature.^[^ ^52^ ^]^ Reactions were carried out with standard Schlenk techniques under a N_2_ atmosphere. To an oven‐dried, N_2_‐purged flask containing 1‐Benzothiophen‐2‐ylboronic acid (1000.0 mg, 5.62 mmol) and 6‐Chlorophenanthridine (1068.3 mg, 5.0 mmol) in toluene/ethanol 100 mL (*v/v* = 2/1), Pd(PPh_3_)_4_ (144.4 mg, 0.125 mmol) was added, followed by the addition of 2 M sodium carbonate aqueous solution (20 mL) under vigorous stirring. The reaction mixture was stirred at 60 °C for 10 h and monitored by TLC. Upon completion, the reaction mixture was washed with brine (2 × 40 mL) and extracted with ethyl acetate (3 × 40 mL). The organic layer was dried over MgSO_4_, and the solvent was evaporated in vacuo to give a crude solid which was purified by column chromatography on silica gel (CH_2_Cl_2_/PE = 1:1) to give **btp** as white solid (1306.2 mg, 4.2 mmol, 84%). **[Ir(piq)_2_(µ‐Cl)]_2_ and [Ir(piq)_2_(µ‐Cl)]_2_**: This compound was synthesized using literature methods.^[^ ^53^ ^]^ In brief, a mixture of IrCl_3_·*n*H_2_O (1.00 g, 2.84 mmol) and piq (1.281 g, 6.25 mmol) or btp (1.950 g, 6.25 mmol) were refluxed in a mixture of 2‐ethoxyethanol and water (100 mL, 3:1, *v/v*) for 24 h. Upon cooling and filtration, the resulting residue was washed with methanol and ether to yield red solids. This dimer can be used in the next reaction without further purification. **[Ir(piq)_2_L] (Ir1)**: **Ir1** was synthesized by reacting **[Ir(piq)_2_(*µ*‐Cl)]_2_** (0.400 g, 0.315 mmol) and **L** (0.248 g, 0.630 mmol) in DCM/MeOH (3/1, 40 mL). The reaction mixture was heated to 50 °C for 5 h and the reaction‐progress was monitored by ESI‐MS. Upon completion, the solvent was evaporated under vacuum to give a crude solid which was purified by flash column chromatography on silica gel (CH_2_Cl_2_ then CH_2_Cl_2_/MeOH = 30/1) to give **Ir1** as a deep purple solid. Yield: 0.501 g (80%). ^1^H NMR (400 MHz, DMSO‐*d*_6_, *δ*) 9.07–8.99 (m, 2H), 8.96 (d, *J* = 9.0 Hz, 1H), 8.86 (s, 1H), 8.83 (d, *J* = 5.7 Hz, 1H), 8.39 (d, *J* = 7.6 Hz, 1H), 8.31 (dd, *J* = 7.6, 4.1 Hz, 2H), 8.22 (d, *J* = 9.0 Hz, 1H), 8.16 (d, *J* = 8.4 Hz, 1H), 8.11–8.04 (m, 2H), 7.97 (d, *J* = 8.9 Hz, 1H), 7.93–7.84 (m, 4H), 7.80–7.71 (m, 3H), 7.67 (d, *J* = 4.2 Hz, 1H), 7.64 (t, *J* = 7.7 Hz, 1H), 7.57 (d, *J* = 6.6 Hz, 1H), 7.49 (d, *J* = 6.5 Hz, 1H), 7.43 (d, *J* = 15.3 Hz, 1H), 7.13–7.06 (m, 2H), 7.02 (s, 1H), 6.86 (t, *J* = 7.0 Hz, 1H), 6.77 (t, *J* = 8.0 Hz, 1H), 6.39 (d, *J* = 7.2 Hz, 1H), 6.20 (d, *J* = 7.7 Hz, 1H), 4.69 (q, *J* = 7.4 Hz, 2H), 2.05 (d, *J* = 13.4 Hz, 6H), 1.46 (t, *J* = 7.2 Hz, 3H). ^13^C NMR (101 MHz, DMSO‐*d*_6_, *δ*) 179.20, 178.52, 168.45, 167.37, 153.33, 151.78, 147.32, 145.89, 145.83, 145.35, 143.40, 140.32, 139.53, 138.48, 136.58, 136.52, 136.38, 134.78, 133.61, 132.72, 132.54, 132.44, 132.27, 131.71, 130.79, 130.24, 130.04, 129.94, 129.34, 129.18, 129.12, 128.18, 127.80, 127.61, 127.04, 126.46, 126.19, 126.07, 125.99, 125.53, 125.51, 122.48, 121.62, 121.48, 121.34, 121.14, 119.40, 115.37, 112.44, 104.26, 52.32, 28.98, 26.83, 26.80, 13.41. ESI‐MS *m/z*: calcd for C_57_H_44_IrN_4_O^+^ [M]^+^: 993.31; found: 993.55. Anal. Calcd for C_57_H_44_IrN_4_O: C 61.12, H 3.96, N 5.00; found: C 60.63, H 4.03, N 4.83. **[Ir(btp)_2_L] (Ir2)**: **Ir2** was synthesized by reacting **[Ir(piq)_2_(*µ*‐Cl)]_2_** (0.400 g, 0.235 mmol) and **L** (185.1 g, 0.471 mmol) in DCM/MeOH (3/1, 40 mL). The reaction mixture was heated to 50 °C for 5 h and the reaction‐progress was monitored by ESI‐MS. Upon completion, the solvent was evaporated under vacuum to give a crude solid which was purified by flash column chromatography on silica gel (CH_2_Cl_2_ then CH_2_Cl_2_/MeOH = 30/1) to give **Ir2** as a deep purple solid. Yield: 0.409 g (72%). ^1^H NMR (400 MHz, DMSO‐*d*_6_, *δ*) 9.34 (t, *J* = 7.9 Hz, 2H), 8.98–8.91 (m, 1H), 8.87 (d, *J* = 8.4 Hz, 1H), 8.68–8.44 (m, 5H), 8.36 (d, *J* = 9.2 Hz, 1H), 8.28–8.02 (m, 9H), 7.92 (dd, *J* = 17.7, 8.6 Hz, 2H), 7.74–7.67 (m, 2H), 7.60 (t, *J* = 7.6 Hz, 1H), 7.42 (dd, *J* = 17.8, 7.9 Hz, 2H), 7.28 (q, *J* = 7.2 Hz, 2H), 7.22 (s, 1H), 7.16 (d, *J* = 15.2 Hz, 1H), 7.11 (t, *J* = 7.5 Hz, 1H), 6.86 (t, *J* = 7.8 Hz, 1H), 6.76 (dd, *J* = 10.1, 4.4 Hz, 3H), 6.59 (t, *J* = 7.6 Hz, 1H), 6.26 (d, *J* = 8.2 Hz, 1H), 4.63–4.47 (m, 2H), 1.88 (d, *J* = 18.5 Hz, 6H), 1.33 (t, *J* = 7.5 Hz, 3H). ^13^C NMR (101 MHz, DMSO‐*d*_6_, *δ*) 179.32, 176.30, 168.03, 166.47, 161.59, 158.71, 146.25, 145.76, 145.56, 145.50, 143.90, 143.68, 143.32, 143.19, 142.84, 138.30, 138.10, 137.55, 136.52, 134.24, 134.20, 133.49, 133.42, 133.24, 133.00, 132.46, 130.99, 130.77, 130.00, 129.48, 129.15, 129.07, 128.45, 128.16, 127.76, 127.52, 127.41, 126.94, 126.90, 126.50, 126.27, 126.14, 125.86, 125.63, 125.30, 124.93, 124.68, 124.40, 123.64, 123.52, 123.43, 123.28, 123.16, 122.70, 122.53, 122.51, 122.30, 121.94, 121.60, 117.83, 114.80, 114.63, 112.43, 104.69, 52.34, 29.04, 29.00, 26.53, 13.31. ESI‐MS *m/z*: calcd for C_69_H_48_IrN_4_OS_2_^+^ [M]^+^: 1205.29; found: 1205.47. Anal. Calcd for C_69_H_48_IrN_4_OS_2_: C 62.20, H 3.63, N 4.21; found: C 61.18, H 4.12, N 4.01.

### UV/Vis Absorption Spectra

UV/vis absorption of **L** (20 µM), **Ir1** (20 µM) and **Ir2** (20 µM) were measured in PBS (pH 7.4), MeOH and CH_2_Cl_2_ at 298 K. The compound tested was dissolved in DMSO just before the experiments, and the final concentration of DMSO was kept at 1% (*v/v*).

### The Generation of ^1^O_2_

**L** (20 µM), **Ir1** (20 µM) or **Ir2** (20 µM) were incubated with ABDA (100 µM) in PBS (pH 7.4), then the mixture was irradiated with 630 nm laser (120 mW cm^−2^, 10 s). The absorption spectrum was scanned and the absorption value of ABDA at 400 nm was recorded. The above operation was repeated for 6 times. For experiments under anoxic conditions, the cuvette solution was argon‐purged for 3 min before measurements; all other procedures followed the aforementioned protocol.

### The Generation of O_2_•^‒^

**L** (20 µM), **Ir1** (20 µM) or **Ir2** (20 µM) was incubated with DHR 123 (10 µM) in PBS (pH 7.4), then the mixture was irradiated with 630 nm laser (120 mW cm^−2^, 10 s). The emission spectra were scanned to record the emission maximum value of DHR123 (*λ*_ex_ = 488 nm, *λ*_em_ = 525 nm). The above operation was repeated for 6 times, total 60 s. For experiments under anoxic conditions, the cuvette solution was argon‐purged for 3 min before measurements; all other procedures followed the aforementioned protocol.

### The Generation of •OH

**L** (20 µM), **Ir1** (20 µM) or **Ir2** (20 µM) was incubated with HPF (10 µM) in PBS (pH 7.4), then the mixture was irradiated with 630 nm laser (120 mW cm^−2^, 10 s). The emission spectra were scanned to record the emission maximum value of HPF (*λ*_ex_ = 490 nm, *λ*_em_ = 515 nm). The above operation was repeated for 6 times, total 60 s. For experiments under anoxic conditions, the cuvette solution was argon‐purged for 3 min before measurements; all other procedures followed the aforementioned protocol.

### Depletion of GSH in Solution

The consumption of GSH was monitored by UV/Vis spectroscopy. **L** (20 µM), **Ir1** (20 µM) or **Ir2** (20 µM was mixed with GSH (120 µM) at room temperature. The mixture was then irradiated with a 630 nm laser (120 mW cm^−2^ for 10 s). Following irradiation, 5,5′‐dithiobis‐(2‐nitrobenzoic acid) (DTNB) (100 µM) was added. After a 5‐minute incubation period, the supernatant was analyzed by UV/Vis spectroscopy, and the absorbance at 412 nm was recorded. This entire procedure was repeated 6 times, total 1 min.

### Photocatalytic Oxidation of NADH by UV/Vis Spectroscopy

The catalytic oxidation of NADH was monitored by UV‐vis spectroscopy. **L** (20 µM), **Ir1** (20 µM) or **Ir2** (20 µM was mixed with NADH (100 µM) at room temperature. then the mixture was irradiated with 630 nm laser (120 mW cm^−2^ for 10 s). The absorption spectrum was scanned to record the absorption value at 339 nm. The above operation was repeated for 6 times, total 60 s.

### Cell Lines and Culture Conditions

The 4T1, HeLa, MC38 and MDA‐MB‐231 cell lines (Experimental Animal Center of Sun Yat‐sen University, Guangzhou, China) were cultured in DMEM (Gibco) containing 10% FBS (Gibco) at 37 °C in a 5% CO_2_ humidified atmosphere or Three‐Gas Hypoxia Incubator (2% O_2_, 5% CO_2_, 93% N_2_).

### Cell Cytotoxicity

For cell viability assessment, cells were seeded in 96‐well plates and cultured for 24 h to allow adherence. Test compounds were subsequently added to each well, adjusting the final volume to complete medium (200 µL). Following 24 h of drug treatment, cells were irradiated with 630 nm light at 120 mW cm^−2^. After an additional 44 h incubation period, the culture medium was carefully removed and replaced with MTT solution (2.5 mg mL^−1^ in PBS, 40 µL). The plates were then incubated for 4 h at 37 °C to allow formazan formation. The resulting formazan crystals were dissolved by adding DMSO (150 µL) to each well, followed by 10 min of orbital shaking (300 rpm) at room temperature. Absorbance was measured at 570 nm with a reference wavelength of 630 nm using a Tecan Infinite M200 Pro microplate reader (Tecan, Switzerland). The resulting absorbance data were utilized to calculate the cell viability inhibition rate. Finally, the half‐maximal inhibitory concentration (IC_50_) was determined by fitting the data using GraphPad Prism 8 software.

### ICP‐MS

After the cells were treated with **Ir1** (5 µM) and **Ir2** (5 µM) for 24 h. Remove the culture medium by aspiration, rinse the cells three times with PBS, detach adherent cells using a cell scraper, collect the cell suspension, and lyse the cells with ice‐cold lysis buffer. The concentration of protein was measured using the BCA Protein Quantitation Kit. Cells were counted and digested in 60% HNO_3_ at 60 °C for 1 h. The solution was then diluted to a final volume of 10 mL with Milli‐Q water. The concentration of iridium was measured using the XSERIES 2 ICP‐MS.

### Flow Cytometry Detection of ROS

MDA‐MB‐231 cells were treated with **Ir1** (0.1 µM) for 24 h, then exposed to 630 nm light irradiation (120 mW cm^−2^) for 1 h. Post irradiation, cells were washed three times with PBS and incubated with DCFH‐DA (10 µM) for 30 min to detect total ROS levels. Fluorescence measurements were performed using excitation at 488 nm and emission at 525 ± 40 nm. For flow cytometric analysis, cells were trypsinized, quenched with serum‐containing medium, and centrifuged at 3,000 rpm for 5 min. The cell pellets were resuspended in PBS (400 µL) and analyzed immediately using a CytoFLEX S flow cytometer (Beckman Coulter) to determine intracellular ROS levels.

### Laser Confocal Microscopy Detection of ROS

After 24 h of incubation with **Ir1** (0.1 µM), MDA‐MB‐231 cells were exposed to 630 nm light irradiation (120 mW cm^−2^) for 1 h. To detect intracellular ROS generation, the cells were subsequently incubated with DCFH‐DA (10 µM) for 30 min (λ_ex_ = 488 nm; λ_em_ = 535 ± 25 nm), followed by three washes with PBS. Fluorescence imaging was then conducted using laser confocal microscopy.

### Mitochondrial Membrane Potential Detection by Laser Confocal Microscopy

Following 24 h incubation with **Ir1** (0.1 µM), MDA‐MB‐231 cells were irradiated with 630 nm light (120 mW cm^−2^) for 1 h. For mitochondrial membrane potential detection, cells were then incubated with JC‐1 (10 µM) probe for 30 min (JC‐1 monomers: λ_ex_ = 514 nm; λ_em_ = 530 ± 20 nm. JC‐1 aggregates: λ_ex_ = 561 nm; λ_em_ = 590 ± 20 nm) and washed three times with PBS. Fluorescence imaging was performed using laser confocal microscopy.

### Mitochondrial Membrane Potential Detection by Flow Cytometry

After 24 h treatment with **Ir1** (0.1 µM), MDA‐MB‐231 cells were subjected to 630 nm light irradiation (120 mW cm^−2^) for 1 h. Mitochondrial membrane potential was assessed using JC‐1 probe (10 µM, 30 min incubation) with the following fluorescence parameters: JC‐1 monomers: λ_ex_ = 514 nm, λ_em_ = 530 ± 20 nm; JC‐1 aggregates: λ_ex_ = 561 nm, λ_em_ = 590 ± 20 nm. Following PBS washes (3 ×), cells were trypsinized, neutralized with serum‐containing medium, and pelleted by centrifugation (3,000 rpm, 5 min). The resulting cell suspension (400 µL PBS) was immediately analyzed for JC‐1 fluorescence ratios using a CytoFLEX S flow cytometer (Beckman Coulter).

### Transmission Electron Microscope

MDA‐MB‐231 cells were first incubated with **Ir1** (0.1 µM) for 24 hours. Subsequently, the cells were irradiated with 630 nm light (120 mW cm^−2^) for 1 hour. After irradiation, the culture medium was removed, and the cells were washed three times with PBS to eliminate residual agents. One hour later, the cells were detached using trypsin, and digestion was terminated by adding serum‐containing medium. The cell suspension was then centrifuged to remove excess medium. For structural preservation, the cells were fixed with electron microscopy fixative at room temperature for 30 minutes and stored at 4 °C. Finally, cellular morphology was examined using a transmission electron microscope (JEM‐2010HR, Japan).

### Western Blot

MDA‐MB‐231 cells were incubated with **Ir1** (0.1 µM) for 24 h, followed by irradiation with 630 nm light (120 mW cm^−2^) for 1 h. After irradiation, the medium was aspirated, and cells were washed three times with PBS. The cells were then harvested in 1.5 mL centrifuge tubes and lysed on ice for 30–50 min using cell lysis buffer (supplemented with PMSF, 60 µL). The lysates were centrifuged at 12000 ×g for 15 min, and the supernatant was collected for protein quantification via an enhanced BCA protein assay kit. The quantified lysates were mixed with dual‐color SDS‐PAGE loading buffer, denatured at 95–100 °C for 10–15 min, and stored at –20 °C. Proteins were separated on 12.5% SDS‐PAGE gels (prepared using an SDS‐PAGE gel kit) at 80 V in running buffer. Proteins were transferred to PVDF membranes at 350 mA for 90 min in transfer buffer. Membranes were blocked with 5% BSA for 40–60 min, then incubated overnight with primary antibody in diluent. After three PBST (PBS + 0.1% Tween 20) washes (5–10 min each), membranes were probed with HRP‐conjugated secondary antibody for 2 h, followed by additional PBST washes. Protein bands were visualized after development.

### Various Death Inhibitors Reverse Cell Viability

Cells were seeded in 96‐well plates and allowed to adhere for 24 h. Test compounds and various death inhibitors (necrostatin‐1, liproxstatin‐1, Z‐VAD‐FMK, 3‐Methyladenine, disulfiram) were then added, adjusting the final volume to 200 µL with complete medium. After 24 h of treatment, cells were irradiated with 630 nm light (120 mW cm^−2^) and incubated for an additional 44 h. For MTT analysis, the medium was replaced with MTT solution (2.5 mg mL^−1^ in PBS, 40 µL), followed by 4 h incubation at 37 °C to allow formazan formation. The crystals were solubilized with DMSO (150 µL) per well and subjected to orbital shaking (300 rpm, 10 min) at room temperature. Absorbance was measured at 570 nm (reference: 630 nm) using a Tecan Infinite M200 Pro microplate reader (Tecan, Switzerland).

### Immunofluorescence (IF) on Cells

MDA‐MB‐231 cells were treated with **Ir1** (0.1 µM) for 24 h, then irradiated with 630 nm light (120 mW cm^−2^) for 1 h. After irradiation, the medium was removed, and cells were washed three times with PBS. Subsequently, cells were fixed with 4% paraformaldehyde (PFA) for 15 min at room temperature, permeabilized with 0.1% Triton X‐100, and blocked with 3% bovine serum albumin (BSA) in PBS for 1 h. For immunofluorescence staining, cells were incubated overnight at 4 °C with primary antibodies against CRT or HMGB1, followed by incubation with a Cy3‐conjugated goat anti‐rabbit IgG secondary antibody (1:200) for 2 h at room temperature. Nuclei were counterstained with DAPI (1 µg mL^−1^, 5 min). Fluorescence images were acquired using a laser scanning confocal microscope with appropriate filter sets for Cy3 (λ_ex_ = 561 nm; λ_em_ = 585 ± 42 nm) and DAPI (λ_ex_ = 405 nm; λ_em_ = 470 ± 50 nm).

### RNA Sequencing

Total RNA was isolated from tissue samples using TRIzol Reagent (Invitrogen) according to the manufacturer's protocol. RNA integrity and purity were rigorously assessed using two complementary approaches: (1) electrophoretic analysis on the 5300 Bioanalyzer system (Agilent Technologies), where only samples with RNA Quality Number (RQN) ≥ 6.5 and 28S:18S ribosomal RNA ratio ≥ 1.0 were retained; and (2) spectrophotometric quantification using the ND‐2000 system (NanoDrop Technologies), requiring samples to meet strict purity criteria (*A*_260_/*A*_280_ = 1.8‐2.2; *A*_260_/*A*_230_ ≥ 2.0) and minimum concentration threshold (>1 µg). Only RNA samples satisfying all quality parameters were processed for downstream library preparation. All RNA sequencing procedures, including library preparation and sequencing, were conducted by Shanghai Majorbio Bio‐pharm Biotechnology Co., Ltd. following standardized protocols. RNA‐seq libraries were prepared from 1 µg high‐quality total RNA using the Illumina Stranded mRNA Prep Kit (San Diego, CA). Briefly, poly(A)+ RNA was enriched using oligo(dT) magnetic beads and chemically fragmented. First‐strand cDNA synthesis was performed using random hexamers with the SuperScript Double‐Stranded cDNA Synthesis Kit (Invitrogen), followed by second‐strand synthesis incorporating dUTP for strand specificity. The resulting cDNA underwent end repair, A‐tailing, and adapter ligation, followed by size selection (∼300 bp) on 2% Low Range Ultra Agarose gels. After 15 cycles of PCR amplification with Phusion High‐Fidelity DNA Polymerase (NEB), library quality was verified by Qubit 4.0 quantification. Final libraries were sequenced on either: (i) Illumina NovaSeq X Plus (PE150) using NovaSeq Reagent Kits, or (ii) MGI DNBSEQ‐T7 (PE150) platform using DNBSEQ‐T7RS Reagent Kit v3.0. Following paired‐end sequencing, raw reads were subjected to stringent quality control and trimming using Fastp with default parameters. Subsequently, the resulting high‐quality reads were aligned to the reference genome in an orientation‐aware manner using HISAT2. For each sample, transcripts were then assembled from the aligned reads using StringTie in a reference‐guided approach. To identify differentially expressed genes (DEGs) between two distinct sample groups, transcript expression levels were quantified using the transcripts per million (TPM) method and gene expression abundance was estimated using RSEM. Differential expression analysis was performed utilizing either DESeq2 or DEGseq. Genes were considered significantly differentially expressed if they met the following criteria: an absolute log_2_ fold change (|log_2_FC|) ≥ 1 and a false discovery rate (FDR) < 0.05 for DESeq2, or an FDR < 0.001 for DEGseq. Subsequently, functional enrichment analysis, including Gene Ontology (GO) and Kyoto Encyclopedia of Genes and Genomes (KEGG) pathway analyses, was conducted to determine whether DEGs were significantly enriched in specific GO terms or metabolic pathways, using a Bonferroni‐corrected P‐value threshold of < 0.05 relative to the whole‐transcriptome background. GO functional enrichment and KEGG pathway analyses were implemented using Goatools and Python SciPy, respectively. The identification of alternative splicing events in the sample was performed using the recently developed rMATS software. The analysis focused exclusively on isoforms that either aligned with the reference or contained novel splice junctions. Splicing variations were categorized into five distinct types: exon inclusion, exon exclusion, alternative 5′ splice sites, alternative 3′ splice sites, and intron retention events.

### Flow Cytometry Detection of CD47 Expression

MDA‐MB‐231 or 4T1 cells were incubated with **Ir1** (0.1 µM) for 24 h, followed by irradiation with 630 nm light (120 mW cm^−2^) for 1 h. After irradiation, the culture medium was aspirated and cells were washed three times with PBS. The cells were then trypsinized, neutralized with complete medium, and centrifuged at 3,000 rpm for 5 min. After centrifugation, cell pellets were resuspended and blocked with 3% BSA in PBS for 1 h at 4 °C. For cell surface marker analysis, cells were stained with APC‐conjugated anti‐mouse CD47 or PE‐conjugated anti‐mouse CD47 antibodies for 30 min at 4 °C in the dark. The cell pellets were resuspended in PBS (400 µL) and analyzed immediately using a CytoFLEX S flow cytometer (Beckman Coulter) to determine CD47 expression.

### Animal Model and Treatment

All animal experiments were conducted in compliance with the ethical guidelines of Sun Yat‐sen University and were approved by the Institutional Animal Care and Use Committee (IACUC Approval No. SYSU‐IACUC‐2025‐000641). Female BALB/c mice (4‐6 weeks old) were obtained from Sun Yat‐sen University and acclimatized for at least 7 days prior to experiments. Animals were housed in standard polycarbonate cages (n = 5 per cage) under controlled environmental conditions (temperature: 21 ± 2 °C; humidity: 50 ± 10%; 12:12 h light‐dark cycle) with ad libitum access to autoclaved food and purified water. All animal welfare and experimental procedures adhered to the guidelines outlined in the National Institutes of Health Guide for the Care and Use of Laboratory Animals, as well as the ethical regulations established by our university. Efforts were made to minimize the number of animals used in the study and to alleviate any potential suffering experienced by the mice. 4T1 murine breast cancer cells were cultured in DMEM supplemented with 10% fetal bovine serum (37 °C, 5% CO_2_). For tumor inoculation, logarithmic‐phase cells were harvested, resuspended in PBS at 1×10⁷ cells/mL, and 100 µL (1×10^6^ cells) was injected subcutaneously into the right axillary region of each mouse. Unilateral transplanted mode: Tumor‐bearing mice were randomized into five treatment groups (n = 5 per group) at specific endpoints. For one cohort, randomization occurred four days post‐inoculation upon formation of palpable tumors; for the other, it occurred on day 14 post‐inoculation, when the tumor volume reached approximately 400 mm^3^. Five treatment groups: (1) Dark control, (2) Light only (630 nm, 120 mW cm^−2^ for 30 min), (3) Dark + **Ir1** (3 mg kg^−1^), (4) Light + **Ir1** (3 mg kg^−1^), and (5) cisplatin control (3 mg kg^−1^). All treatments were administered via intratumoral injection as a single dose. Tumor volumes were measured every other day (days 1–11 or days 1–7) using calipers and calculated as V = (a × b^2^)/2, where a and b represent the longest and perpendicular diameters, respectively. Mice were euthanized on day 7 or 11 for tumor excision and analysis. Tumor rechallenge model: Four days post‐inoculation when palpable tumors formed, mice were randomly allocated into five treatment groups (n = 5 per group): (1) Dark control, (2) Light only (630 nm, 120 mW cm^−^^2^ for 30 min), (3) Dark + **Ir1** (1 mg kg^−1^), (4) Light + **Ir1** (1 mg kg^−1^), and (5) cisplatin control (3 mg kg^−1^). All treatments were administered via intratumoral injection as a single dose. On day 7, a secondary challenge of 3×10^6^ cells (100 µL) were injected into the left axillary region to assess metastatic potential. Tumor growth was monitored until day 17, followed by euthanasia and tumor collection.

### Tumor Tissue Flow Cytometry

Excised tumor tissue was minced into small fragments in serum‐free digestion (2 mL) solution and transferred to a 15 mL centrifuge tube. After adding digestion solution (3 mL), the mixture was incubated at 37 °C for 2 h with continuous shaking. Digestion was terminated by adding serum‐containing medium. The resulting suspension was filtered through a cell strainer and centrifuged at 1000 rpm for 5 min. The pellet was resuspended in 3–5 volumes of red blood cell lysis buffer, gently pipetted, and incubated for 10 min at room temperature. After centrifugation (1000 rpm, 5 min), the supernatant was discarded. To minimize nonspecific antibody binding, cells were incubated with TruStain Fc^TM^ (anti‐mouse CD16/32) on ice for 40 min. Cells were stained with the following fluorescently labeled antibodies for surface marker detection: APC anti‐mouse CD80, PE anti‐mouse CD86, FITC anti‐mouse CD11c (dendritic cell markers); FITC anti‐mouse CD3, PE anti‐mouse CD4, APC anti‐mouse CD8a (T cell subsets); Alexa Fluor 700 anti‐mouse CD45 (pan‐leukocyte marker); FITC anti‐mouse CD206 (M2 macrophage marker). After 30 min of incubation in the dark, cells were washed once with PBS (6000 rpm, 1 min) and resuspended in PBS for analysis. Flow cytometry was performed using a CytoFLEX S instrument (Beckman Coulter), and data were analyzed with CytExpert software.

### Immunofluorescence or Immunohistochemistry of Mouse Tumor

Mouse tumor specimens were dissected, and the central portion was excised and fixed in 4% paraformaldehyde (PFA). The fixed tissues were then embedded in paraffin and sectioned using a microtome to generate unstained sections, which were stored for long‐term preservation until immunofluorescence analysis. The paraffin‐embedded tissue sections were dried in an electric constant‐temperature blast oven at 70 °C for 2 h. Dewaxing and rehydration were performed as follows: first in xylene (12 minutes per wash, repeated three times), followed by sequential washes in 100% ethanol, 75% ethanol and 50% ethanol (each for 12 minutes per wash, repeated three times), and finally in tap water and double distilled water (each for 10 minutes). The sections were subjected to heat‐induced antigen retrieval in 10 mM citrate buffer (pH 6.0) at 95–100 °C for 20–30 min. After cooling, the sections were rinsed in PBS for 5 min. Permeabilization was performed by incubating the tissue in 0.5% Triton X‐100 for 15 min. Following permeabilization, excess liquid was removed by spin‐drying, and the sections were outlined with a hydrophobic barrier (crayon) to prevent antibody spillage. Non‐specific binding was blocked with 3% normal sheep serum for 2 h at room temperature. The primary antibody (targeting the protein of interest) was applied and incubated overnight at 4 °C. After three PBS washes (5 min each), the sections were incubated with a fluorescent secondary antibody for 2 h at room temperature (protected from light). The sections were washed 3 × with PBS and then stained with DAPI (2 min). Excess DAPI was removed by additional PBS washes (3 ×). Finally, the sections were mounted with 10 µL of antifade mounting medium. Fluorescence images were acquired using a microscope for further analysis.

### Statistical Analysis

All data are presented as mean ± SEM. Statistical significance was determined using one‐way ANOVA followed by Dunnett's post hoc test for multiple comparisons with the control group. A *p* value < 0.05 was considered statistically significant.

## Conflict of Interest

The authors declare no conflict of interest.

## Supporting information



Supporting Information

## Data Availability

The data that support the findings of this study are available from the corresponding author upon reasonable request.

## References

[1] C. Huang , X. Wang , Y. Wang , Y. Feng , X. Wang , S. Chen , P. Yan , J. Liao , Q. Zhang , C. Mao , Y. Li , L. Wang , X. Wang , W. Yi , W. Cai , S. Chen , N. Hong , W. He , J. Chen , W. Jin , Nat. Cancer 2024, 5, 500. 38200243 10.1038/s43018-023-00691-z

[2] Z. Tang , M.‐C. Zhong , J. Qian , C. C. Galindo , D. Davidson , J. Li , Y. Zhao , E. Hui , A. Veillette , Nat. Immunol. 2023, 24, 2032. 37945822 10.1038/s41590-023-01671-2 PMC11750626

[3] Y. Nishiga , A. P. Drainas , M. Baron , D. Bhattacharya , A. A. Barkal , Y. Ahrari , R. Mancusi , J. B. Ross , N. Takahashi , A. Thomas , M. Diehn , I. L. Weissman , E. E. Graves , J. Sage , Nat. Cancer 2022, 3, 1351. 36411318 10.1038/s43018-022-00456-0 PMC9701141

[4] S. A. Yamada‐Hunter , J. Theruvath , B. J. McIntosh , K. A. Freitas , F. Lin , M. T. Radosevich , A. Leruste , S. Dhingra , N. Martinez‐Velez , P. Xu , J. Huang , A. Delaidelli , M. H. Desai , Z. Good , R. Polak , A. May , L. Labanieh , J. Bjelajac , T. Murty , Z. Ehlinger , C. W. Mount , Y. Chen , S. Heitzeneder , K. D. Marjon , A. Banuelos , O. Khan , S. L. Wasserman , J. Y. Spiegel , S. Fernandez‐Pol , C. J. Kuo , et al., Nature 2024, 630, 457. 38750365 10.1038/s41586-024-07443-8 PMC11168929

[5] Y. Li , J. Liu , W. Chen , W. Wang , F. Yang , X. Liu , Y. Sheng , K. Du , M. He , X. Lyu , H. Li , L. Zhao , Z. Wei , F. Wang , S. Zheng , J. Sui , J. Hematol. Oncol. 2023, 2, 10.1186/s13045-023-01399-4. PMC9844003 36650558

[6] J. C. Osorio , P. Smith , D. A. Knorr , J. V. Ravetch , Cancer Cell 2023, 41, 2051e2056. 37977147 10.1016/j.ccell.2023.10.007 PMC10842210

[7] C. Grandclément , C. Estoppey , E. Dheilly , M. Panagopoulou , T. Monney , C. Dreyfus , J. Loyau , V. Labanca , A. Drake , S. De Angelis , A. Rubod , J. Frei , L. N. Caro , S. Blein , E. Martini , M. Chimen , T. Matthes , Z. Kaya , C. M. Edwards , J. R. Edwards , E. Menoret , C. Kervoelen , C. Pellat‐Deceunynck , P. Moreau , M. L. Mbow , A. Srivastava , M. R. Dyson , E. A. Zhukovsky , M. Perro , S. Sammicheli , Nat. Commun. 2024, 15, 2054. 38448430 10.1038/s41467-024-46310-y PMC10917784

[8] B. R. Schrank , Y. Wang , A. Wu , N. Tran , D. Lee , J. Edwards , K. Huntoon , S. Dong , J. Ha , Y. Ma , A. J. Grippin , S. D. Jeong , A. Antony , M. Chang , M. Kang , T. D. Gallup , A. C. Koong , J. Li , K. Yun , B. Y. S. Kim , W. Jiang , Nat. Cancer 2025, 6, 511. 40000910 10.1038/s43018-025-00919-0 PMC11952976

[9] R. Shi , Y. Chai , X. Duan , X. Bi , Q. Huang , Q. Wang , S. Tan , G. F. Gao , J. Zhu , J. Yan , Signal Transduct. Targeted Ther. 2020, 5, 16. 10.1038/s41392-020-0121-2 PMC7058617 32296041

[10] D. Xie , Y. Tian , D. Hu , Y. Wang , Y. Yang , B. Zhou , R. Zhang , Z. Ren , M. Liu , J. Xu , C. Dong , B. Zhao , L. Yang , Signal Transduct. Targeted Ther. 2023, 8, 436. 10.1038/s41392-023-01683-2 PMC10684539 38016957

[11] J. Yang , Y. Song , K. Zhou , Z. Li , M. Zhang , H. Jing , Z. Wang , L. Yu , W. Meng , Q. Lu , W. Tian , Y. Shi , J. Hematol. Oncol. 2024, 17, 123. 39696680 10.1186/s13045-024-01646-2 PMC11657391

[12] J. Yu , S. Li , D. Chen , D. Liu , H. Guo , C. Yang , W. Zhang , L. Zhang , G. Zhao , X. Tu , L. Peng , S. Liu , X. Bai , Y. Song , Z. Jiang , R. Zhang , W. Tian , J. Hematol. Oncol. 2022, 15, 167, 10.1186/s13045-022-01385-2. 36384978 PMC9670587

[13] Z. Liu , H. Chen , N. Ta , Z. Shi , L. Zhan , T. Han , J. Zhang , X. Chang , K. Yin , M. Nie , J. Cancer 2023, 14, 350. 36860925 10.7150/jca.80725 PMC9969583

[14] D. Candas‐Green , B. Xie , J. Huang , M. Fan , A. Wang , C. Menaa , Y. Zhang , L. Zhang , D. Jing , S. Azghadi , W. Zhou , L. Liu , N. Jiang , T. Li , T. Gao , C. Sweeney , R. Shen , T.‐y. Lin , C.‐x. Pan , O. M. Ozpiskin , G. Woloschak , D. J. Grdina , A. T. Vaughan , J. M. Wang , S. Xia , A. M. Monjazeb , W. J. Murphy , L.‐Q. Sun , H.‐W. Chen , K. S. Lam , et al., Nat. Commun. 2020, 11, 4591. 32929084 10.1038/s41467-020-18245-7 PMC7490264

[15] P. Dai , Y. Sun , Z. Huang , Y. T. Liu , M. Gao , H. M. Liu , J. Shi , C. He , B. Xiang , Y. Yao , H. Yu , G. Xu , L. Kong , X. Xiao , X. Wang , X. Zhang , W. Xiong , J. Hu , D. Lin , B. Zhong , G. Chen , Y. Gong , C. Xie , J. Zhang , Nat. Commun. 2025, 16, 4564. 40379682 10.1038/s41467-025-59621-5 PMC12084640

[16] Y. Fan , S. Song , Y. Li , S. S. Dhar , J. Jin , K. Yoshimura , X. Yao , R. Wang , A. W. Scott , M. P. Pizzi , J. Wu , L. Ma , G. A. Calin , S. Hanash , L. Wang , M. Curran , J. A. Ajani , Cancer Res. 2023, 83, 3726. 37738407 10.1158/0008-5472.CAN-23-0783 PMC10843008

[17] J. Theruvath , M. Menard , B. A. H. Smith , M. H. Linde , G. L. Coles , G. N. Dalton , W. Wu , L. Kiru , A. Delaidelli , E. Sotillo , J. L. Silberstein , A. C. Geraghty , A. Banuelos , M. T. Radosevich , S. Dhingra , S. Heitzeneder , A. Tousley , J. Lattin , P. Xu , J. Huang , N. Nasholm , A. He , T. C. Kuo , E. R. B. Sangalang , J. Pons , A. Barkal , R. E. Brewer , K. D. Marjon , J. G. Vilches‐Moure , P. L. Marshall , et al., Nat. Med. 2022, 28, 333. 35027753 10.1038/s41591-021-01625-x PMC9098186

[18] Q.‐F. Meng , Y. Zhao , C. Dong , L. Liu , Y. Pan , J. Lai , Z. Liu , G.‐T. Yu , X. Chen , L. Rao , Angew. Chem., Int. Ed. 2021, 60, 26320. 10.1002/anie.202108342 34661332

[19] S. Moon , M. Jung , S. Go , J. Hong , H. S. Sohn , C. Kim , M. Kang , B. J. Lee , J. Kim , J. Lim , B.‐S. Kim , Adv. Mater. 2024, 36, 2410340. 10.1002/adma.202410340 39252658

[20] Y. e. Liu , Y. Wang , Y. Yang , L. Weng , Q. Wu , J. Zhang , P. Zhao , L. Fang , Y. Shi , P. Wang , Signal Transduct. Targeted Ther. 2023, 8, 104. 10.1038/s41392-023-01365-z PMC9990587 36882399

[21] S. Chen , A. Saeed , Q. Liu , Q. Jiang , H. Xu , G. G. Xiao , L. Rao , Y. Duo , Signal Transduct. Targeted Ther. 2023, 8, 207. 10.1038/s41392-023-01452-1 PMC10200802 37211559

[22] R. Jin , L. Neufeld , T. L. McGaha , Nat. Cancer 2025, 6, 239. 39962208 10.1038/s43018-025-00909-2

[23] L. Tang , Y. Yin , Y. Cao , C. Fu , H. Liu , J. Feng , W. Wang , X. J. Liang , Adv. Mater. 2023, 35, 2303835. 10.1002/adma.202303835 37384818

[24] H. You , S. Zhang , Y. Zhang , Q. Chen , Y. Wu , Z. Zhou , Z. Zhao , B. Su , X. Li , Y. Guo , Y. Chen , W. Tang , B. Liu , H. Fan , S. Geng , M. Fang , F. Li , G. Liu , C. Jiang , T. Sun , Adv. Mater. 2025, 37, 2418053. 10.1002/adma.202418053 40035513

[25] Z. Su , S. Dong , Y. Chen , T. Huang , B. Qin , Q. Yang , X. Jiang , C. Zou , Adv. Sci. 2023, 10, 2206213. 10.1002/advs.202206213 PMC10369263 37132609

[26] W. Lin , Y. Liu , J. Wang , Z. Zhao , K. Lu , H. Meng , R. Luoliu , X. He , J. Shen , Z.‐W. Mao , W. Xia , Angew. Chem., Int. Ed. 2023, 135, 202310158. 10.1002/anie.202310158 37668526

[27] X. Xiong , K.‐B. Huang , Y. Wang , B. Cao , Y. Luo , H. Chen , Y. Yang , Y. Long , M. Liu , A. S. C. Chan , H. Liang , T. Zou , J. Am. Chem. Soc. 2022, 144, 10407. 35658433 10.1021/jacs.2c02435

[28] Y.‐L. Zeng , L.‐Y. Liu , T.‐Z. Ma , Y. Liu , B. Liu , W. Liu , Q.‐H. Shen , C. Wu , Z.‐W. Mao , Angew. Chem., Int. Ed. 2024, 64, 202410803.

[29] X. Li , X. Zhao , X. Wang , A. Xiong , Z. Wang , Z. Shi , J. Zhang , H. Wang , W. Wei , C. He , J. Ma , Z. Guo , C. Duan , J. Zhao , X. Wang , Angew. Chem., Int. Ed. 2025, 64, 202419292. 10.1002/anie.202419292 39673540

[30] L. Zhang , N. Montesdeoca , J. Karges , H. Xiao , Angew. Chem., Int. Ed. 2023, 62, 202300662. 10.1002/anie.202300662 36807420

[31] D. Tang , M. Cui , B. Wang , C. Xu , Z. Cao , J. Guo , H. Xiao , K. Shang , Adv. Mater. 2024, 36, 2406815. 10.1002/adma.202406815 39081102

[32] Y. Lu , F.‐Y. Wang , M. S. Levine , H.‐R. Shi , Y. Wang , X. Xiong , L.‐M. Yang , Y.‐Q. Shi , T. Zou , J. L. Sessler , H. Liang , K.‐B. Huang , J. Am. Chem. Soc. 2025, 147, 15216. 40279467 10.1021/jacs.5c00255

[33] X. Zhu , S. Li , Molecular Cancer 2023, 22, 94. 37312116 10.1186/s12943-023-01797-9 PMC10262535

[34] A. Chaudhary , A. Kumar , N. Swain , K. Chaudhary , H. Sonker , S. Dewan , R. A. Patil , R. G. Singh , Small 2025, 21, 2406809. 10.1002/smll.202406809 39607393

[35] H. Hu , J. Chen , F. Zhang , Z. Sheng , Y. Yang , Y. Xie , L. Zhou , Y. Liu , J. Med. Chem. 2024, 67, 16195. 39264254 10.1021/acs.jmedchem.4c01026

[36] L. Ke , F. Wei , L. Xie , J. Karges , Y. Chen , L. Ji , H. Chao , Angew. Chem., Int. Ed. 2022, 61, 202205429. 10.1002/anie.202205429 35532958

[37] Y. Pei , Y. Pan , Z. Zhang , J. Zhu , Y. Sun , Q. Zhang , D. Zhu , G. Li , M. R. Bryce , D. Wang , B. Z. Tang , Adv. Sci. 2025, 12, 2413879. 10.1002/advs.202413879 PMC11984874 39951332

[38] L. Wang , J. Karges , F. Wei , L. Xie , Z. Chen , G. Gasser , L. Ji , H. Chao , Chem. Sci. 2023, 14, 1461. 36794192 10.1039/d2sc06675k PMC9906708

[39] C. You , L. Tian , J. Zhu , L. Wang , B. Z. Tang , D. Wang , J. Am. Chem. Soc. 2025, 147, 2010. 39763433 10.1021/jacs.4c15150

[40] X. Zhao , J. Liu , J. Fan , H. Chao , X. Peng , Chem. Soc. Rev. 2021, 50, 4185. 33527104 10.1039/d0cs00173b

[41] J. Karges , Angew. Chem., Int. Ed. 2022, 61, 202112236.

[42] Y. Wan , L.‐H. Fu , C. Li , J. Lin , P. Huang , Adv. Mater. 2021, 33, 2103978. 10.1002/adma.202103978 34580926

[43] M. Tavakkoli Yaraki , B. Liu , Y. N. Tan , Nano‐Micro Lett. 2022, 14, 123. 10.1007/s40820-022-00856-y PMC9072609 35513555

[44] Y. Wang , E. Stancliffe , R. Fowle‐Grider , R. Wang , C. Wang , M. Schwaiger‐Haber , L. P. Shriver , G. J. Patti , Mol. Cell 2022, 82, 3270. 35973426 10.1016/j.molcel.2022.07.007 PMC10134440

[45] M. Li , Y. Xu , Z. Pu , T. Xiong , H. Huang , S. Long , S. Son , L. Yu , N. Singh , Y. Tong , J. L. Sessler , X. Peng , J. S. Kim , Proc. Natl. Acad. Sci. U. S. A. 2022, 119, 2210504119. 10.1073/pnas.2210504119 PMC9407644 35969782

[46] P. He , M. Jia , L. Yang , H. Zhang , R. Chen , W. Yao , Y. Pan , Q. Fan , W. Hu , W. Huang , Adv. Mater. 2025, 37, 2418978. 10.1002/adma.202418978 39924790

[47] D. Li , G. Wen , H. Wang , Q. Ren , D. Wang , A. Dao , H. Huang , P. Zhang , J. Med. Chem. 2025, 68, 3749. 39854246 10.1021/acs.jmedchem.4c02836

[48] C. Liu , J. Yan , M. Wu , W. Wang , L. Yuan , D. Tian , Y. Sun , R. Zhang , J. Am. Chem. Soc. 2025, 147, 27812. 40700638 10.1021/jacs.5c06657

[49] F. Chen , H. Ma , G. Wen , X. Wu , X. Lin , D. Li , D. Wang , A. Dao , H. Huang , P. Zhang , J. Med. Chem. 2025, 68, 13019. 40523197 10.1021/acs.jmedchem.5c01006

[50] X. Niu , L. Chen , Y. Li , Z. Hu , F. He , Semin. Cancer Biol. 2022, 86, 273. 35288298 10.1016/j.semcancer.2022.03.009

[51] P. Catsoulis , S. Wang , C. Yelleswarapu , J. Rochford , Photochem. Photobiol. 2023, 99, 547. 36103615 10.1111/php.13715

[52] F. Liu , J. Wen , S.‐S. Chen , S. Sun , Chem. Commun. 2018, 54, 1371. 10.1039/c7cc09723a 29354829

[53] C. Li , M. Yu , Y. Sun , Y. Wu , C. Huang , F. Li , J. Am. Chem. Soc. 2011, 133, 11231. 21682270 10.1021/ja202344c

