# Supplementary material for: Ir(III) Complexes Convert Cold to Hot Tumors via Ferroptosis/Necroptosis‐Driven Immunogenic Cell Death and Photosensitized CD47 Downregulation
Source: Adv Sci (Weinh). 2025 Nov 26;13(8):e14256. doi: 10.1002/advs.202514256 (PMC12884794; doi:10.1002/advs.202514256)
Supplement: Supplementary file 1 — Supporting Information [file ADVS-13-e14256-s001.docx]

Supporting Information

**Ir(III) Complexes Convert Cold to Hot Tumors via Ferroptosis/Necroptosis-Driven Immunogenic Cell Death and Photosensitized CD47 Downregulation**

*Long-Bo Yu,**^†^ Peng Wang,^†^ Qing-Hua Shen, Qi-Xin Guan, Zhi-Yuan Li, Ying-Ying Han, Xin-Yi Zhang, Qing-Yuan Hu, and Cai-Ping Tan**

**Supporting Scheme, Figures and Tables**

**Scheme S1.** Synthetic schemes of **L**, **Ir1** and **Ir2**.


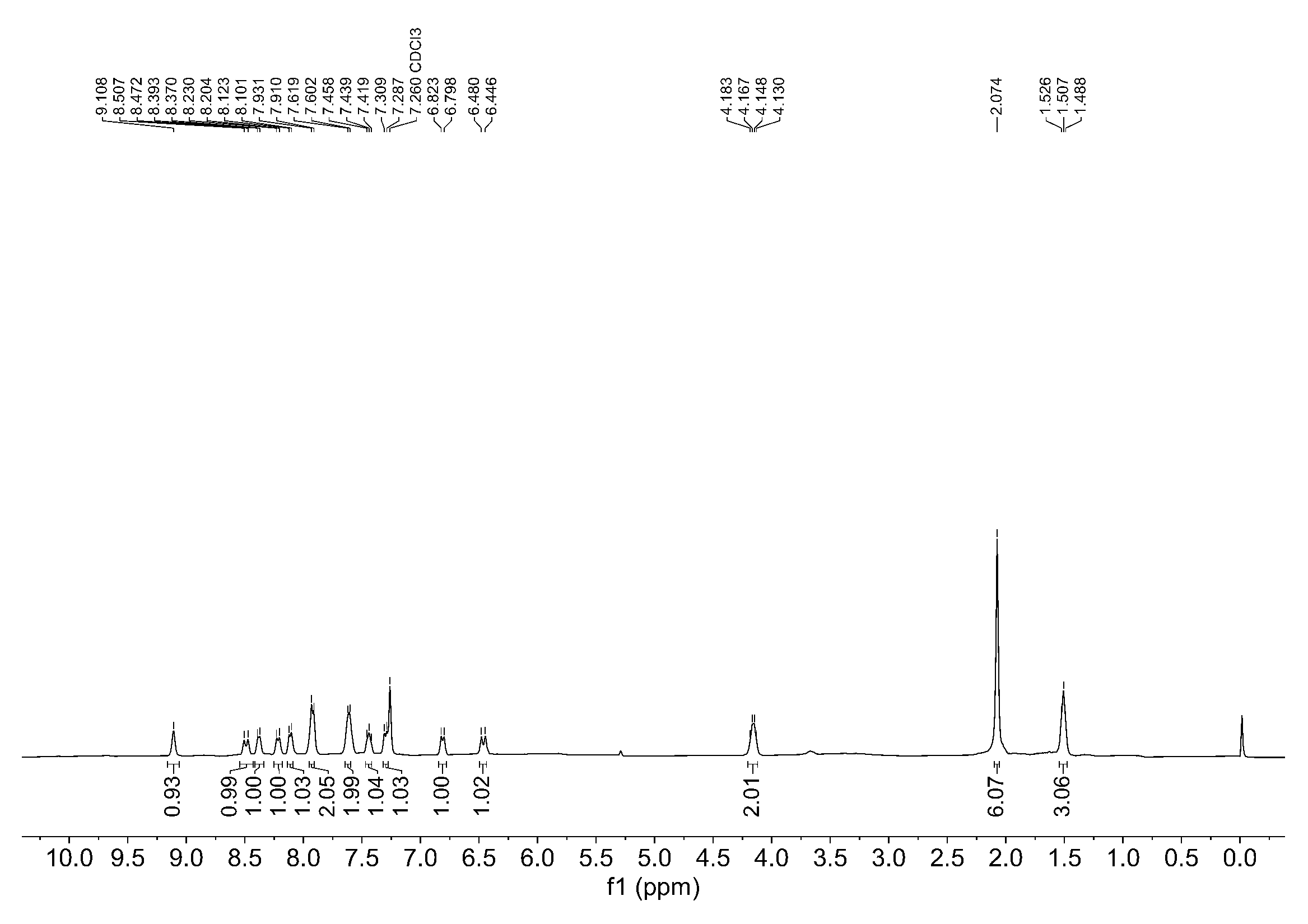


**Figure S1.** ^1^H NMR spectrum of **L** in Chloroform-*d*.


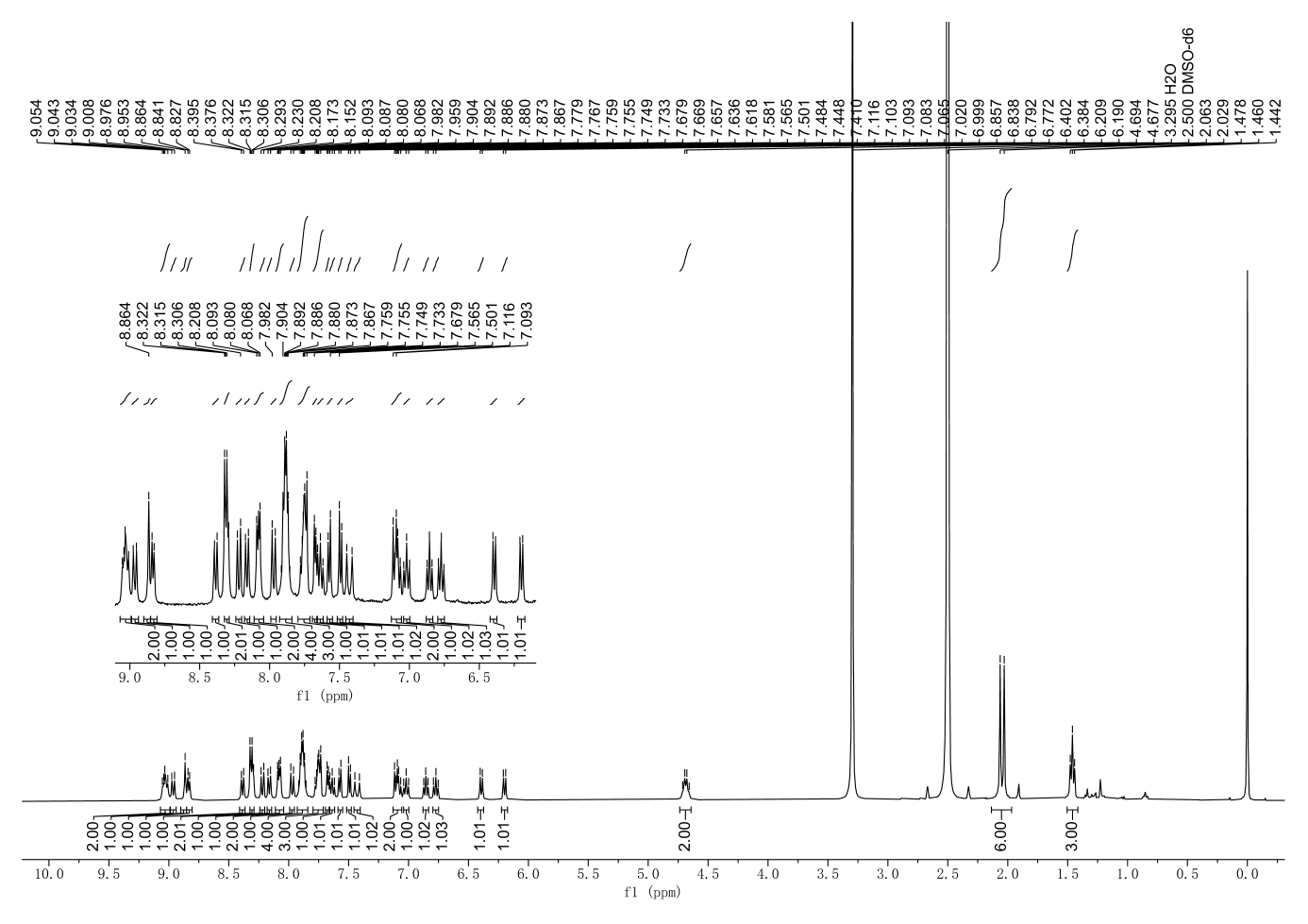


**Figure S2.** ^1^H NMR spectrum of **Ir1** in DMSO-*d*_6_.


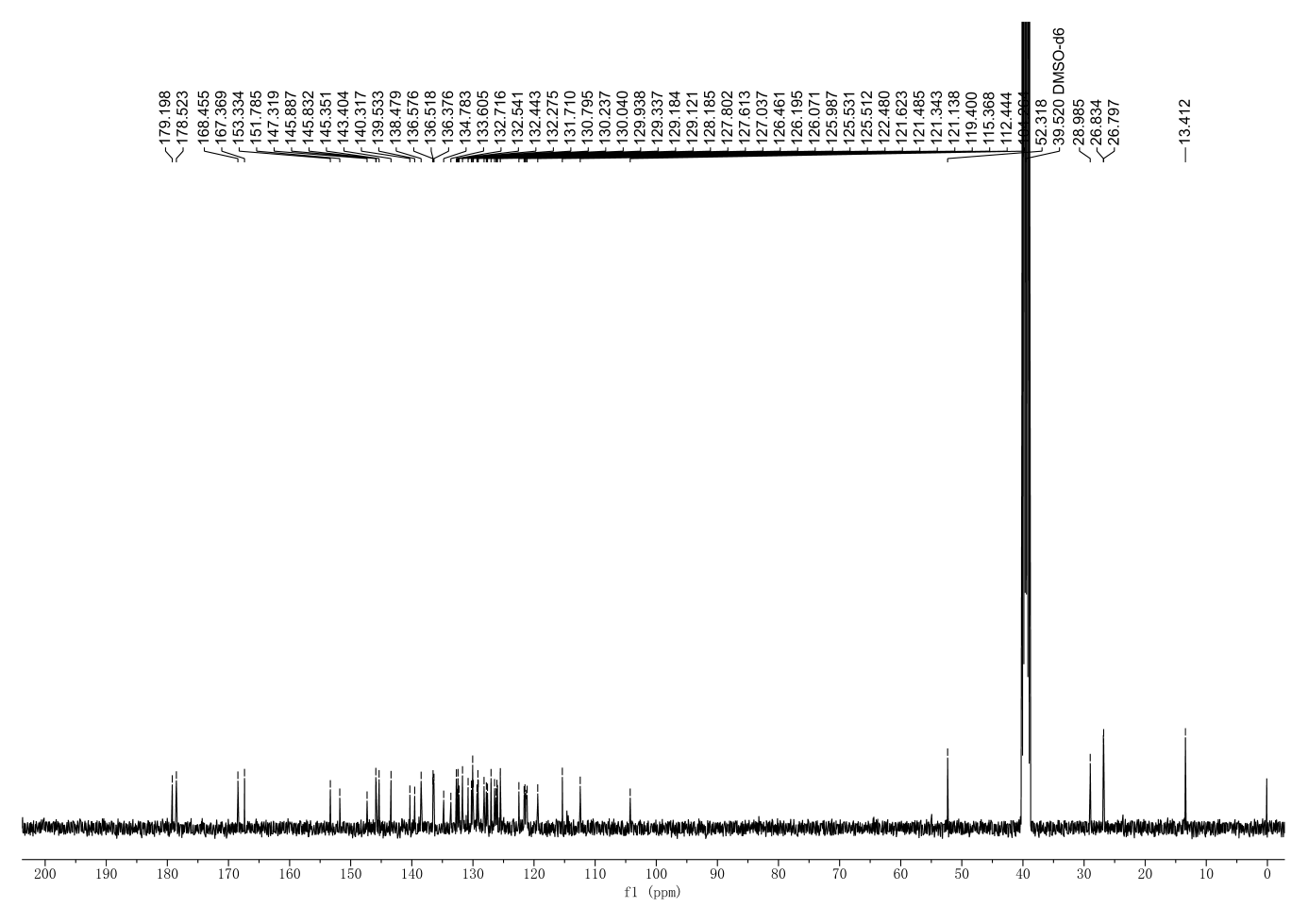


**Figure S3.** ^13^C NMR spectrum of **Ir1** in DMSO-*d*_6_.

**Figure S4.** ESI-MS spectrum of **Ir1** in methanol.


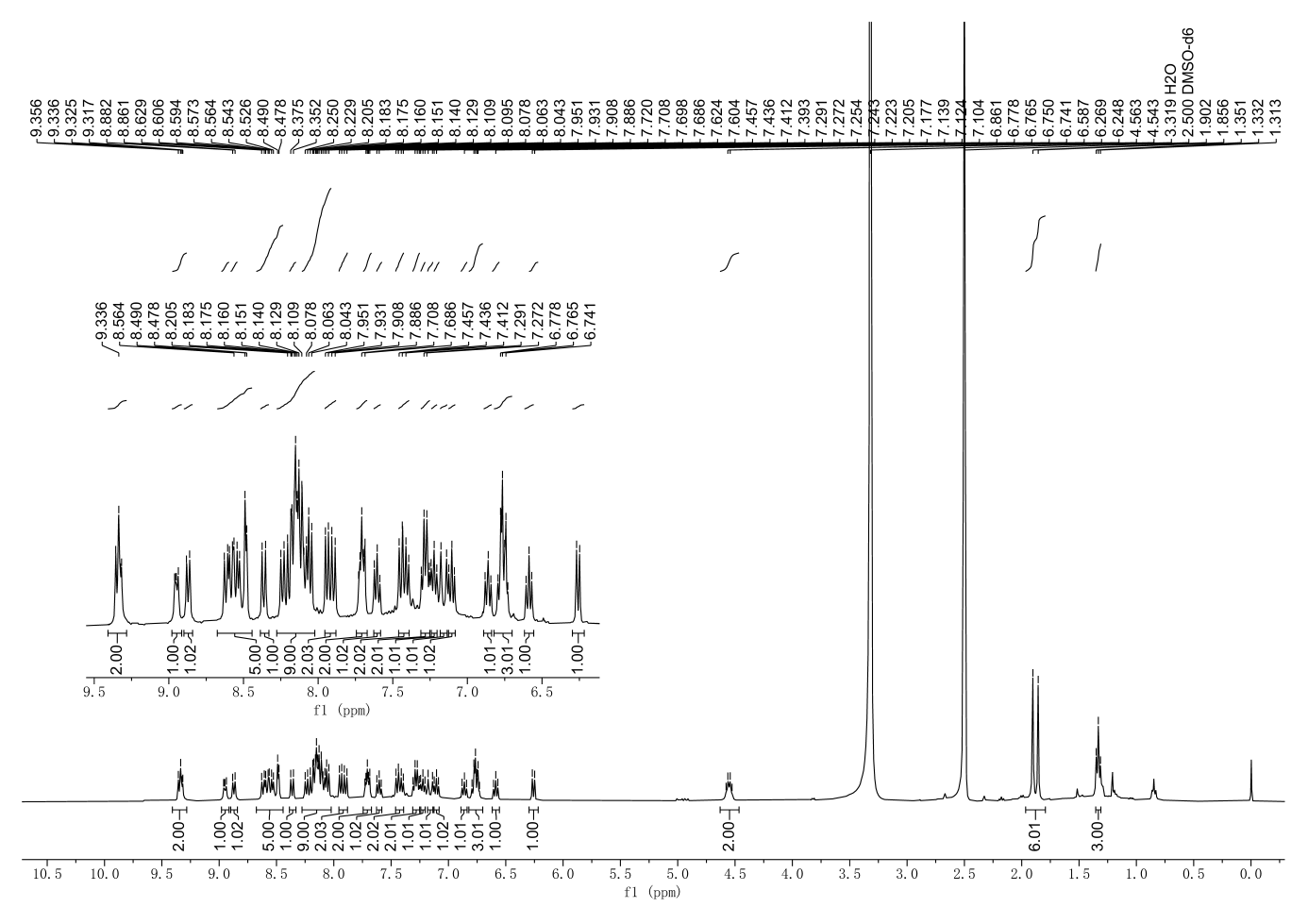


**Figure S5.** ^1^H NMR spectrum of **Ir2** in DMSO-*d*_6_.


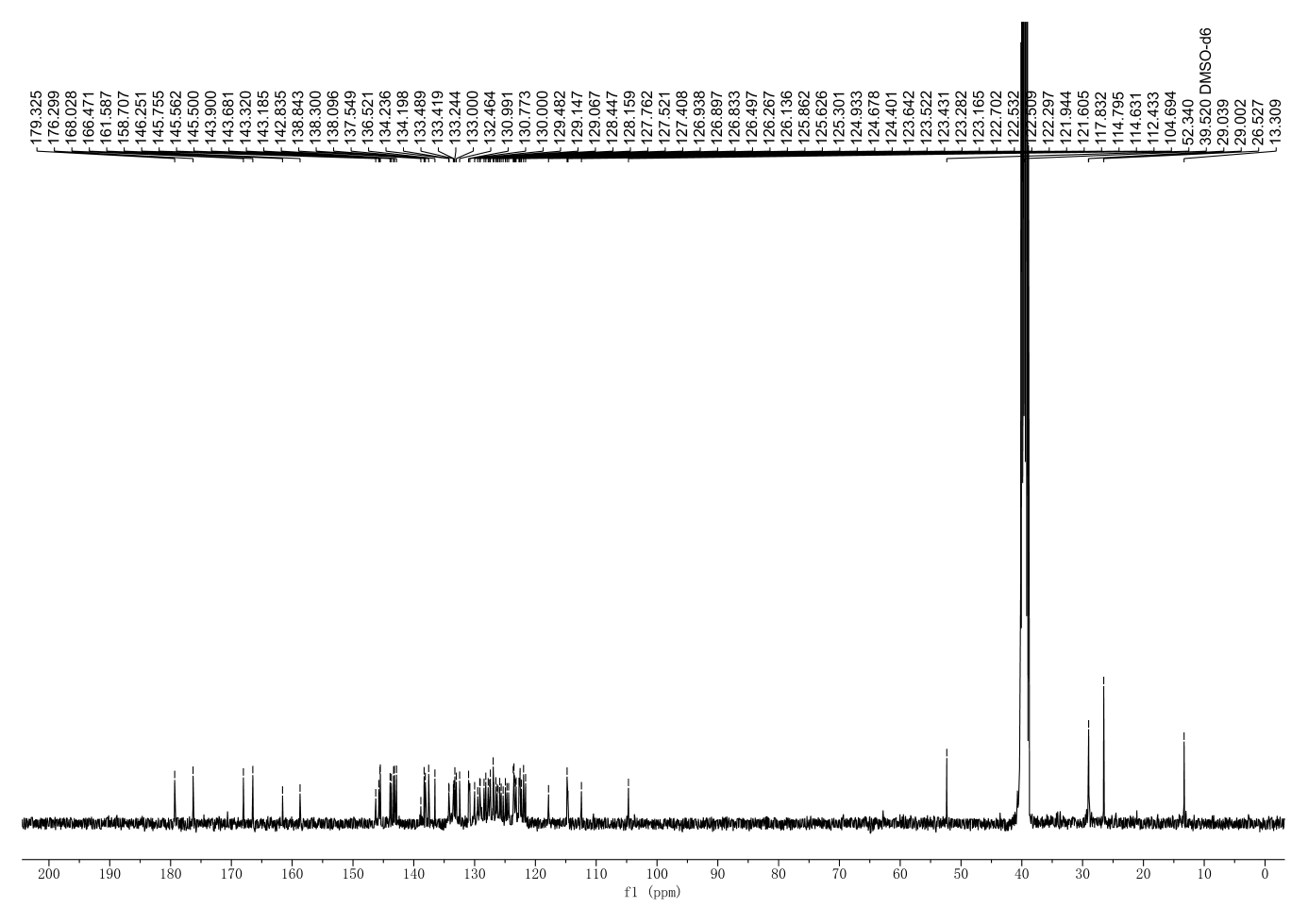


**Figure S6.** ^13^C NMR spectrum of **Ir2** in DMSO-*d*_6_.

**Figure S7.** ESI-MS spectrum of **Ir2** in methanol.


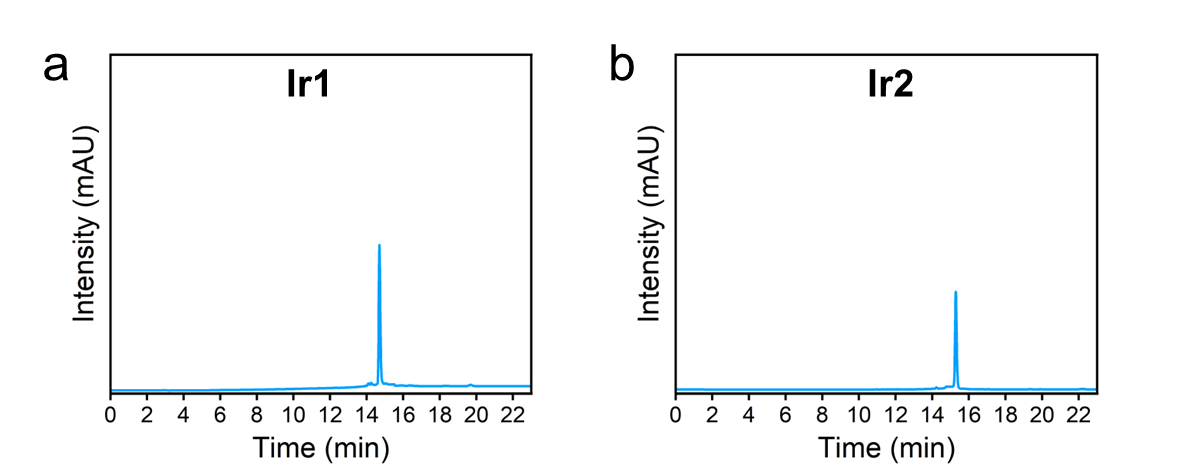


**Figure S8.** HPLC analysis of **Ir1** and **Ir2** (C_18_ column, MeOH/H_2_O (95/5, *v/v*)).


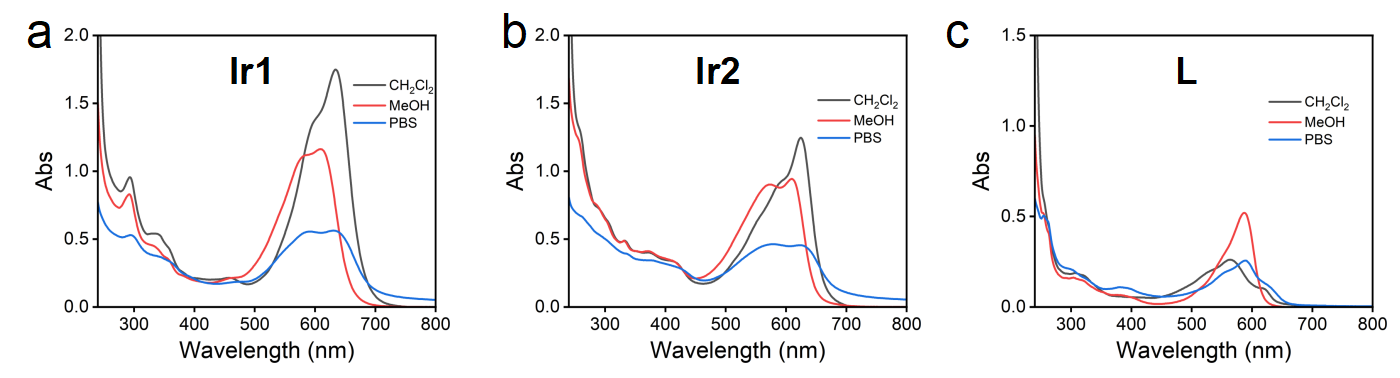


**Figure S9.** UV/Vis absorption spectra of **Ir1** (20 μM), **L** (20 μM) and **Ir2** (20 μM) in PBS, CH_2_Cl_2_ and MeOH.


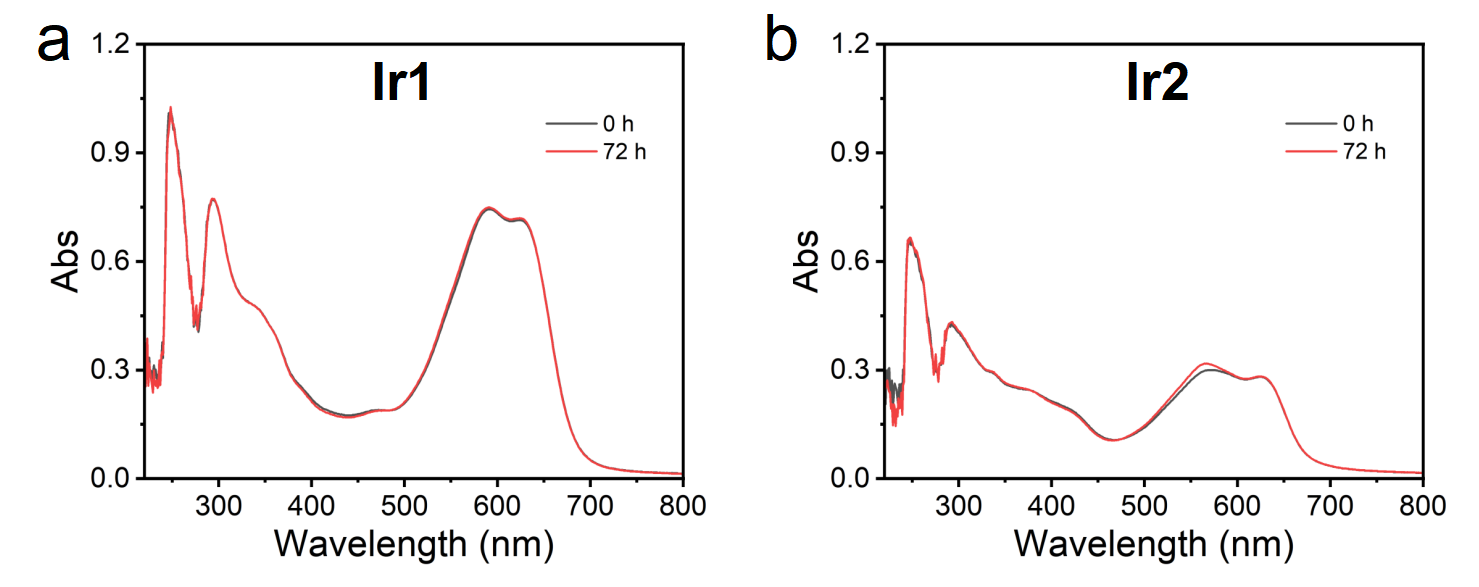


**Figure S10.** UV/Vis spectra of **Ir1** (a) and **Ir2** (b) in DMEM (with 10% FBS) for 72 h and 0 h.


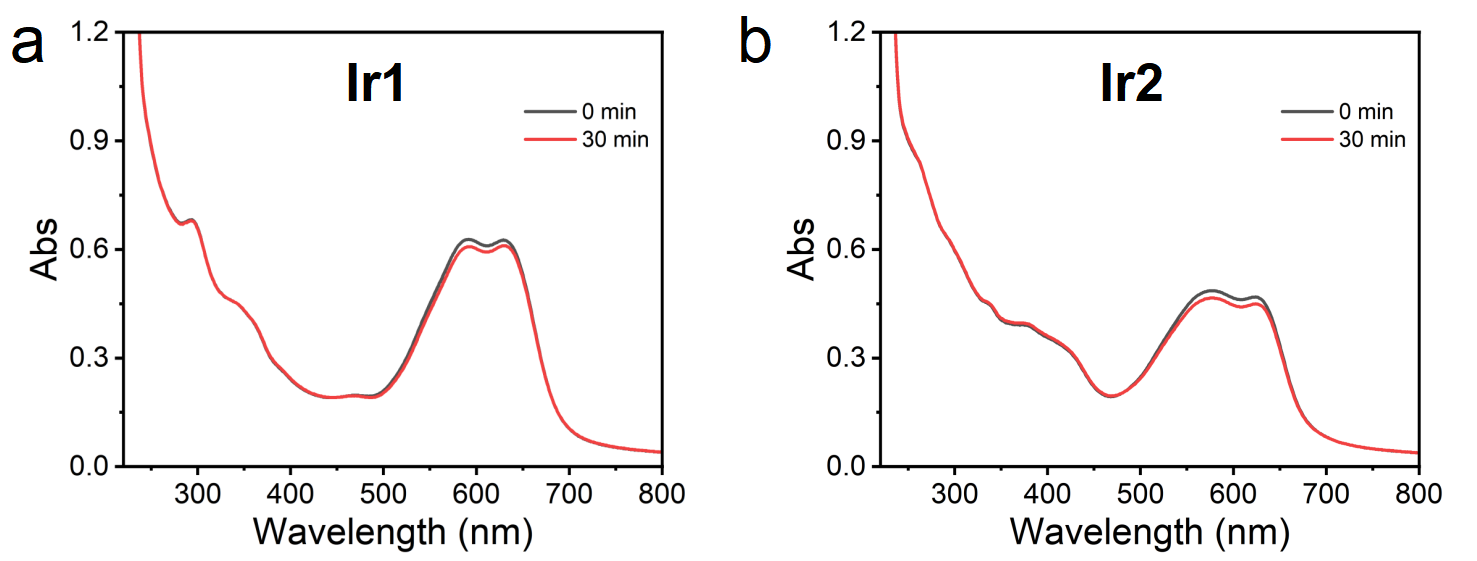


**Figure S11.** UV/Vis spectra of **Ir1** (a) and **Ir2** (b) under 630 nm light irradiation for 30 min and dark conditions.


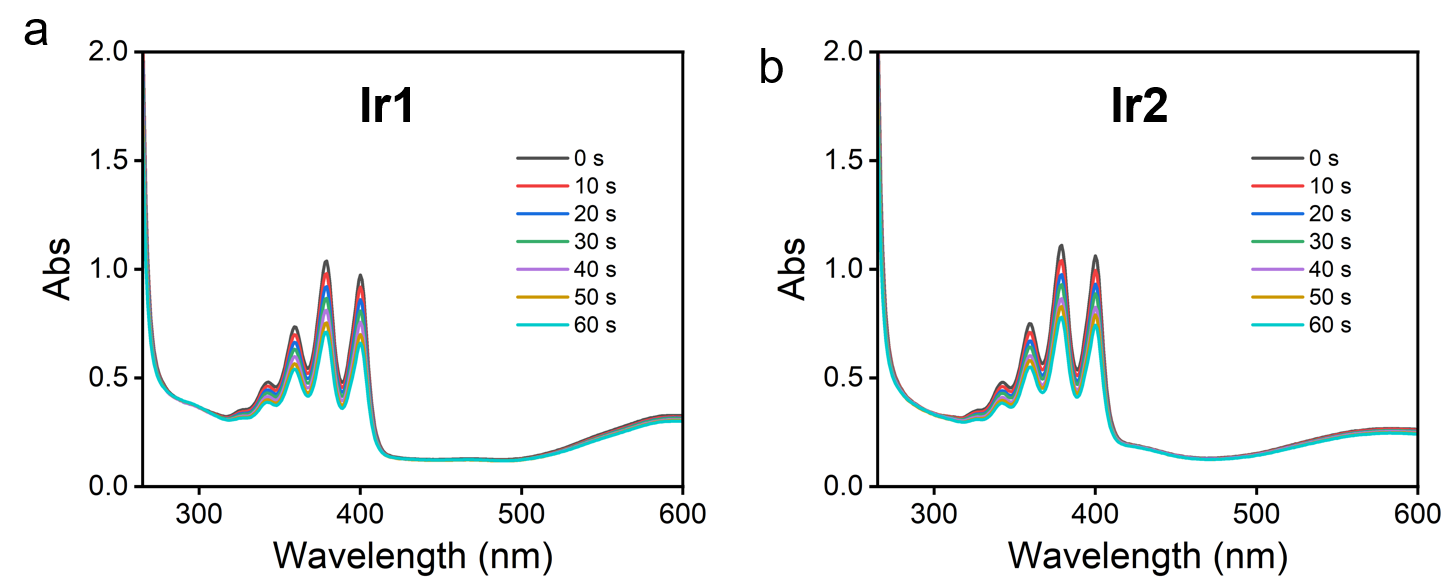


**Figure S12.** The changes in the UV/vis absorption spectra of ABDA (100 µM) incubated with **Ir1** (20 µM) and **Ir2** (20 μM) upon light irradiation (120 mW cm^‒2^, 0–60 s) under hypoxia.


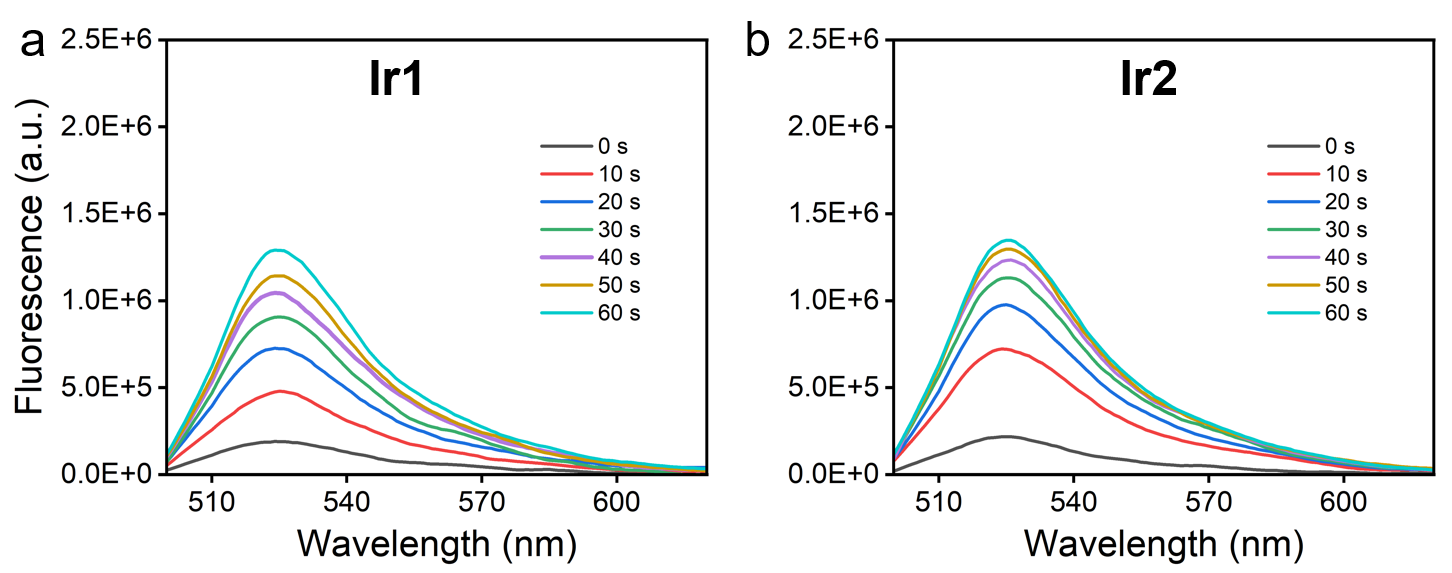


**Figure S13.** The emission spectra of DHR 123 (10 µM, λ_ex_ = 488 nm) incubated with **Ir1** (20 µM) and **Ir2** (20 μM) upon light irradiation (120 mW cm^−2^, 0–60 s) under hypoxia.


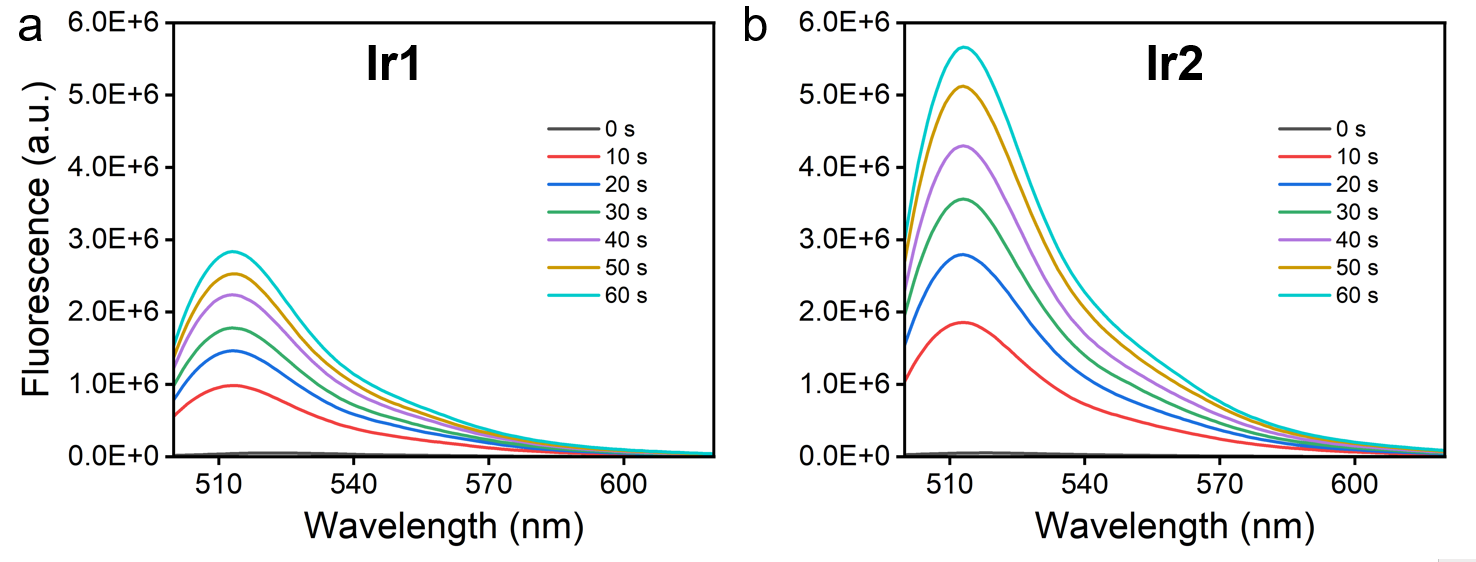


**Figure S14.** The emission spectra of HPF (10 µM, λ_ex_ = 490 nm) incubated with **Ir1** (20 µM) and **Ir2** (20 μM) upon light irradiation (120 mW cm^−2^, 0–60 s) under hypoxia.


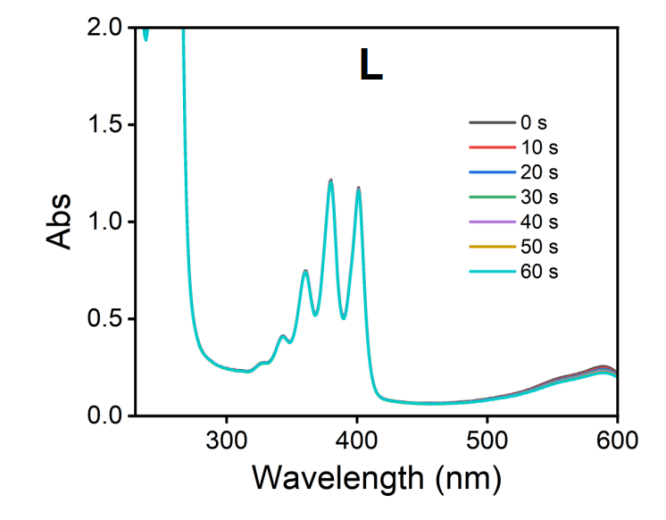


**Figure S15.** The changes in the UV/vis absorption spectra of ABDA (100 µM) incubated with **L** (20 µM) upon light irradiation (120 mW cm^‒2^, 0–60 s) under normoxia.


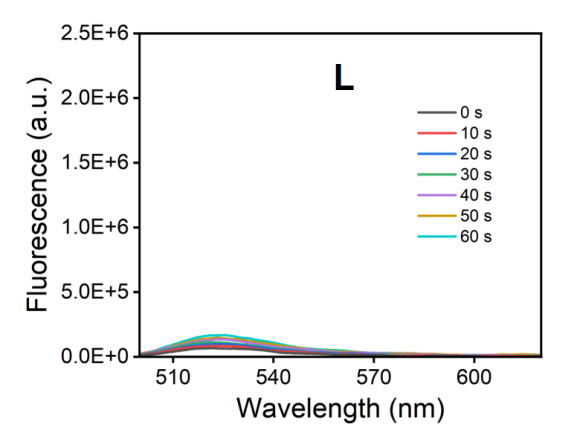


**Figure S16.** The emission spectra of DHR 123 (10 µM, λ_ex_ = 488 nm) incubated with **L** (20 µM) upon light irradiation (120 mW cm^−2^, 0–60 s) under normoxia.


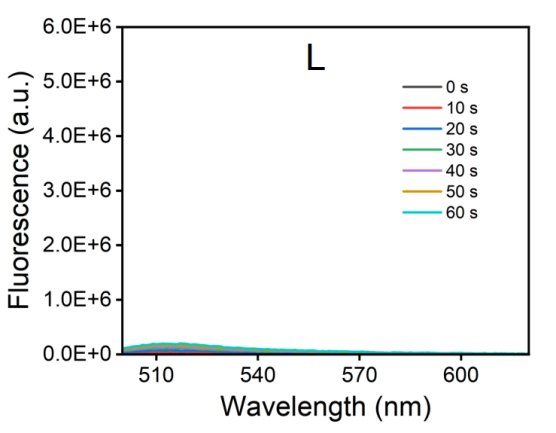


**Figure S17.** The emission spectra of HPF (10 µM, λ_ex_ = 490 nm) incubated with **Ir1** (20 µM) upon light irradiation (120 mW cm^−2^, 0–60 s) under normoxia.


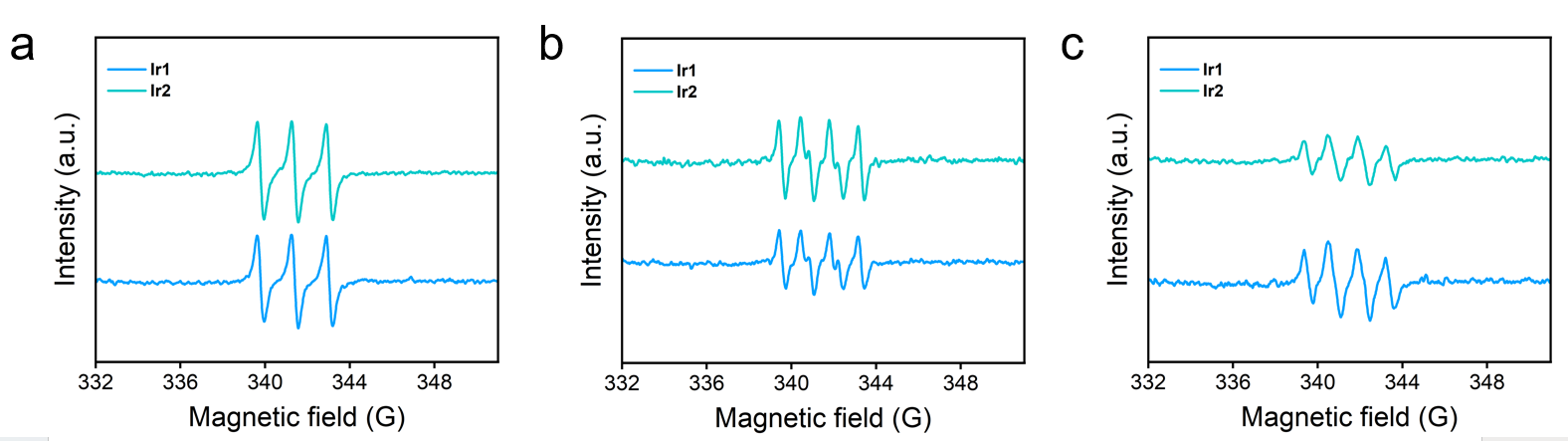


**Figure S18.** (a) The ESR signal of TMPO (0.5 mM) in the presence of **Ir1** (0.5 mM) and **Ir2** (0.5 mM) upon light irradiation (120 mW cm^−2^, 60 s). (b) The ESR signal of DMPO in the presence of **Ir1** (0.5 mM) and **Ir2** (0.5 mM) upon light irradiation (120 mW cm^−2^, 60 s). (c) The ESR signal of DMPO in the presence of **Ir1** (0.5 mM) and **Ir2** (0.5 mM) upon light irradiation (120 mW cm^−2^, 60 s).


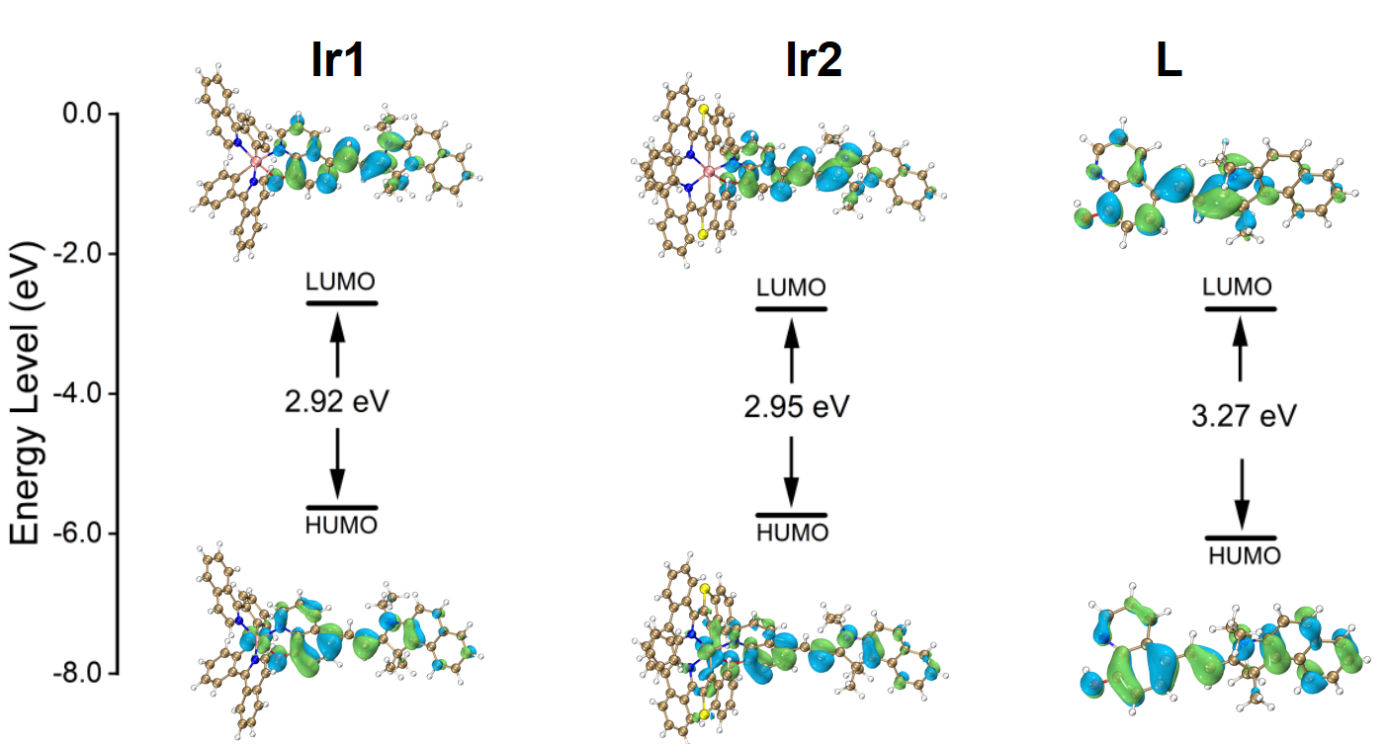


**Figure S19.** Frontier molecular orbital energy levels of **Ir1**, **Ir2**, and **L** in the T1 state.


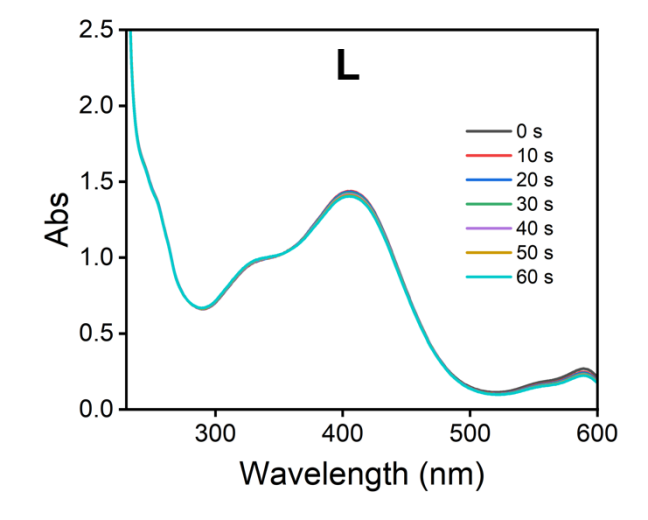


**Figure S20.** The consumption of GSH (100 μM) monitored by DTNB (100 μM) of **L** upon light irradiation (120 mW cm^−2^, 60 s).


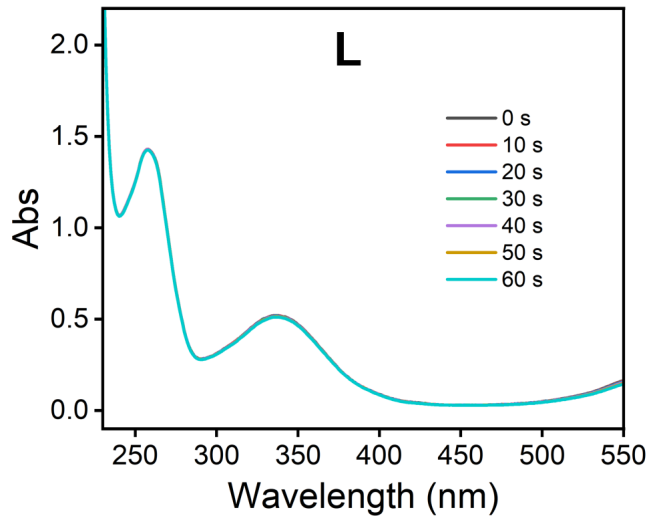


**Figure S21.** The changes in the UV/Vis absorption spectra of NADH (100 μM) incubated with **L** (20 µM).


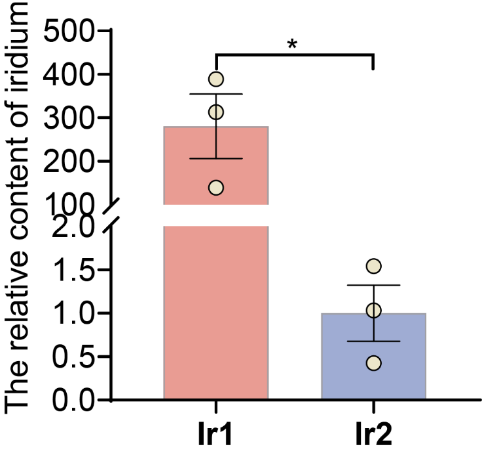


**Figure S22.** ICP-MS measured the intracellular iridium concentration in cells treated for 24 hours with **Ir1** (5 µM) or **Ir2** (5 µM).


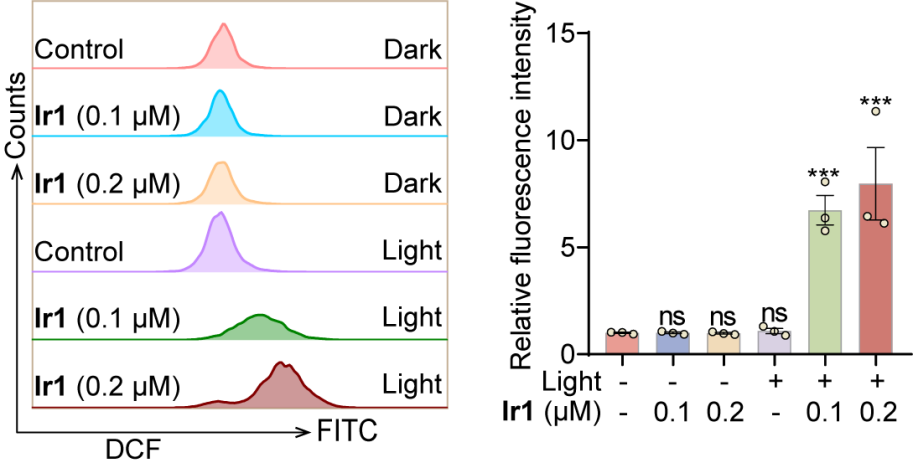


**Figure S23.** Impact of **Ir1** on ROS levels measured by DCF (10 μM, 30 min) staining using flow cytometry under hypoxia.


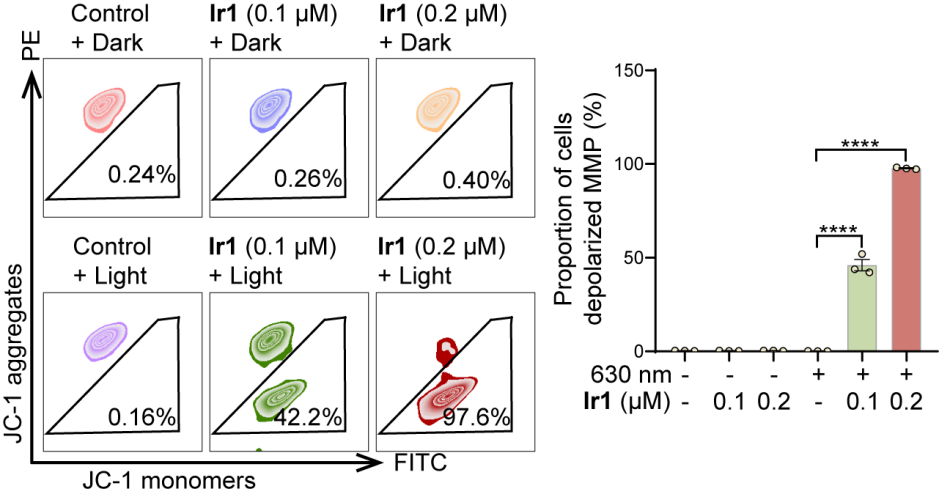


**Figure S24.** Impact of **Ir1**-mediated PDT on MMP using flow cytometry (JC-1 monomers: λ_ex_ = 488 nm; λ_em_ = 525 ± 40 nm. JC-1 aggregates: λ_ex_ = 561 nm; λ_em_ = 585 ± 42 nm) under hypoxia.


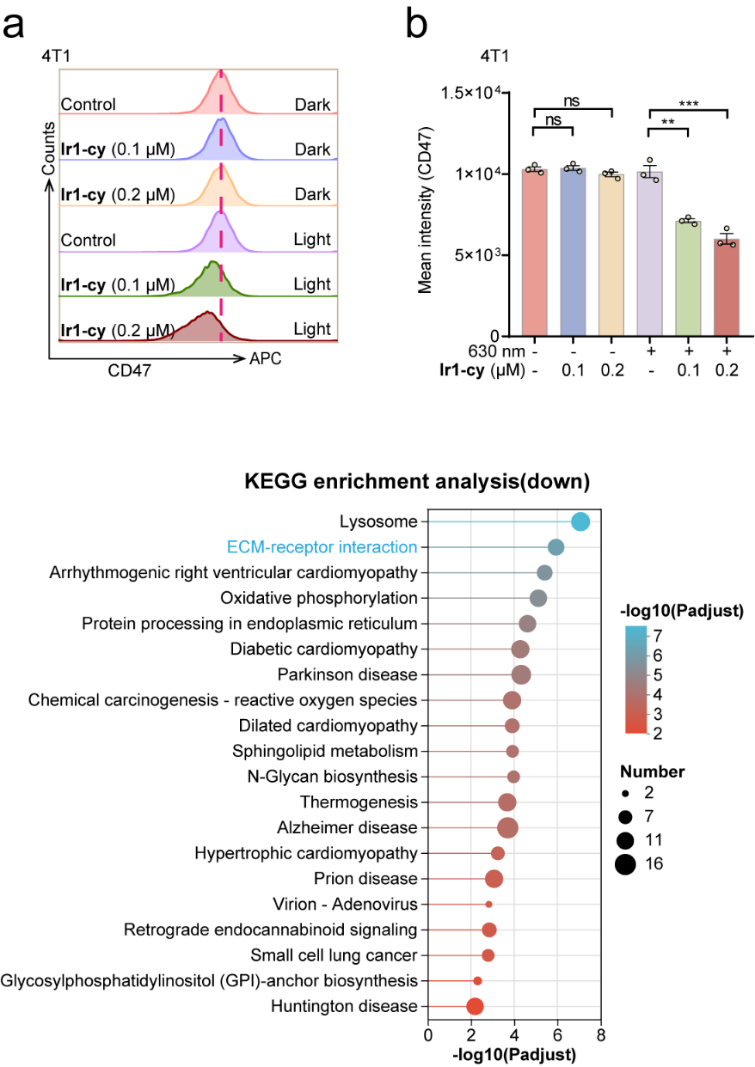


**Figure S25.** KEGG pathway enrichment of down-regulated genes. Pathways are ordered by increasing adjusted *p*-value (top 20 shown).


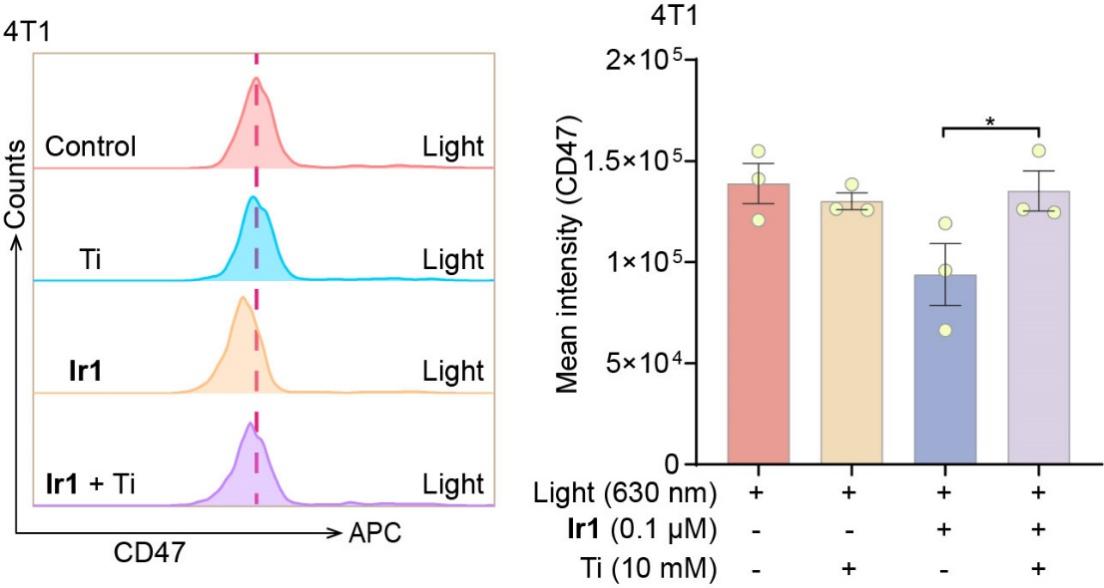


**Figure S26.** Flow cytometric analysis of CD47 expression. **Ir1**: 0.1 μM, n = 3.


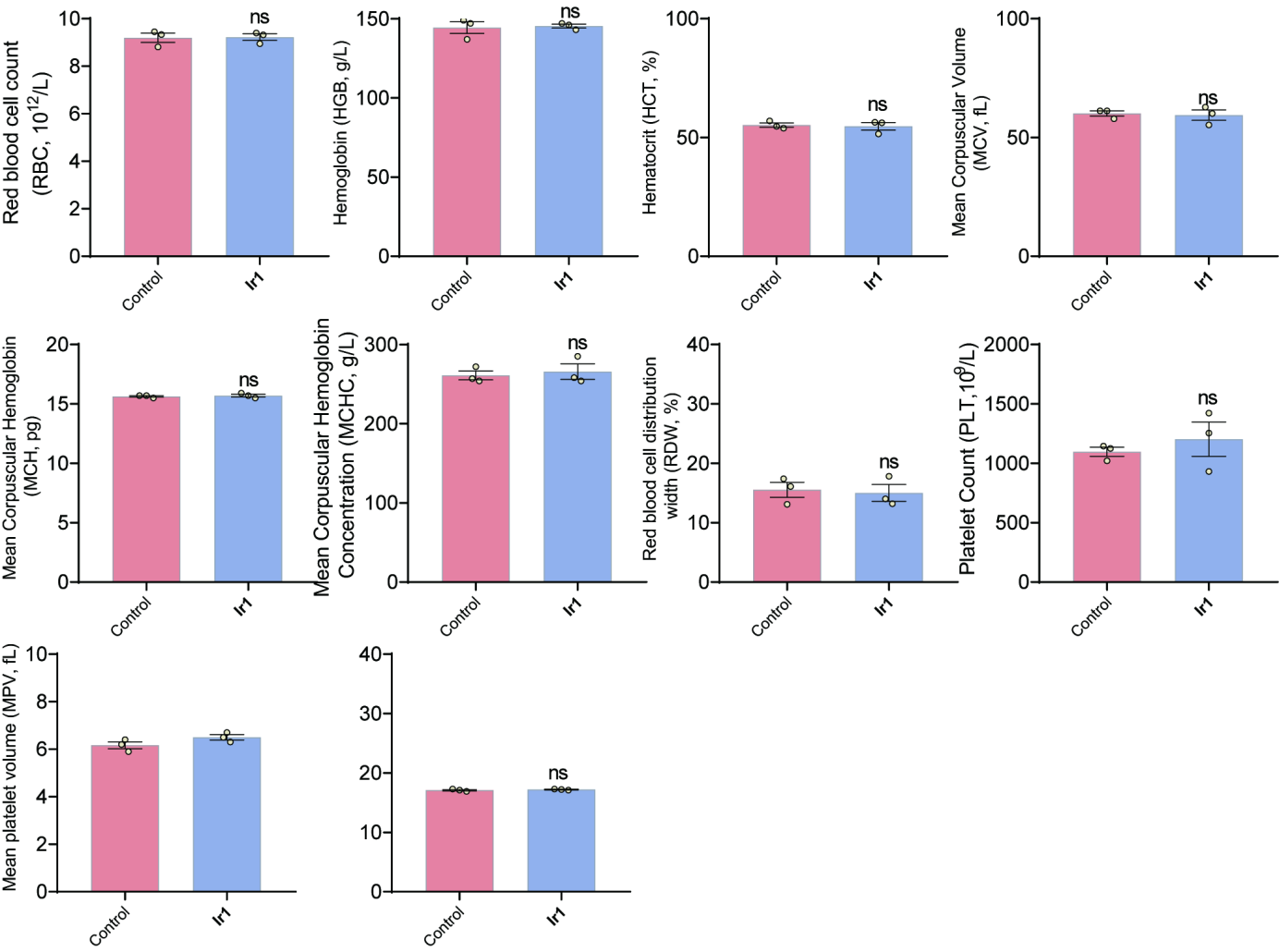


**Figure S27.** Analysis of red blood cells and platelets.


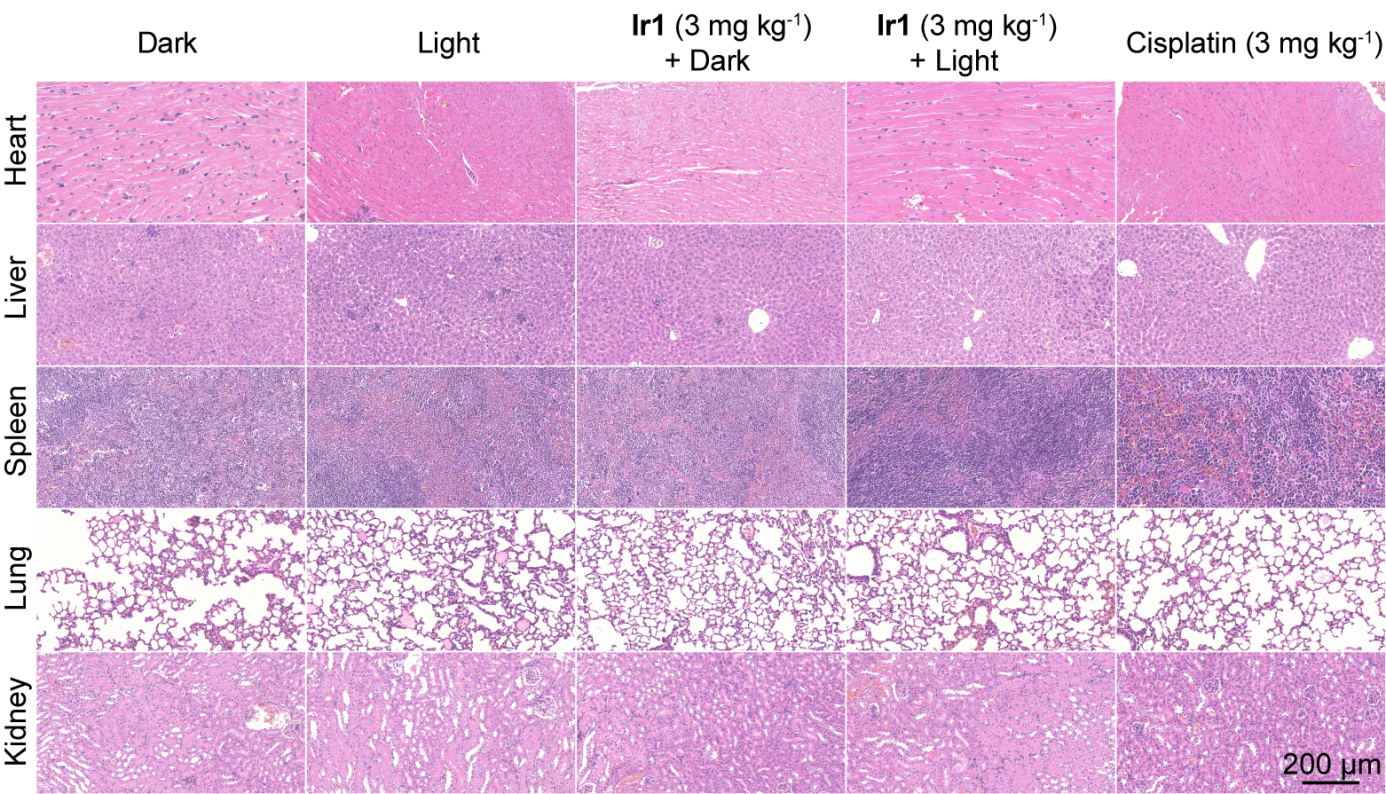


**Figure S28.** H&E sections of mouse heart, liver, spleen, lung and kidney. Scale bar: 200 μm.


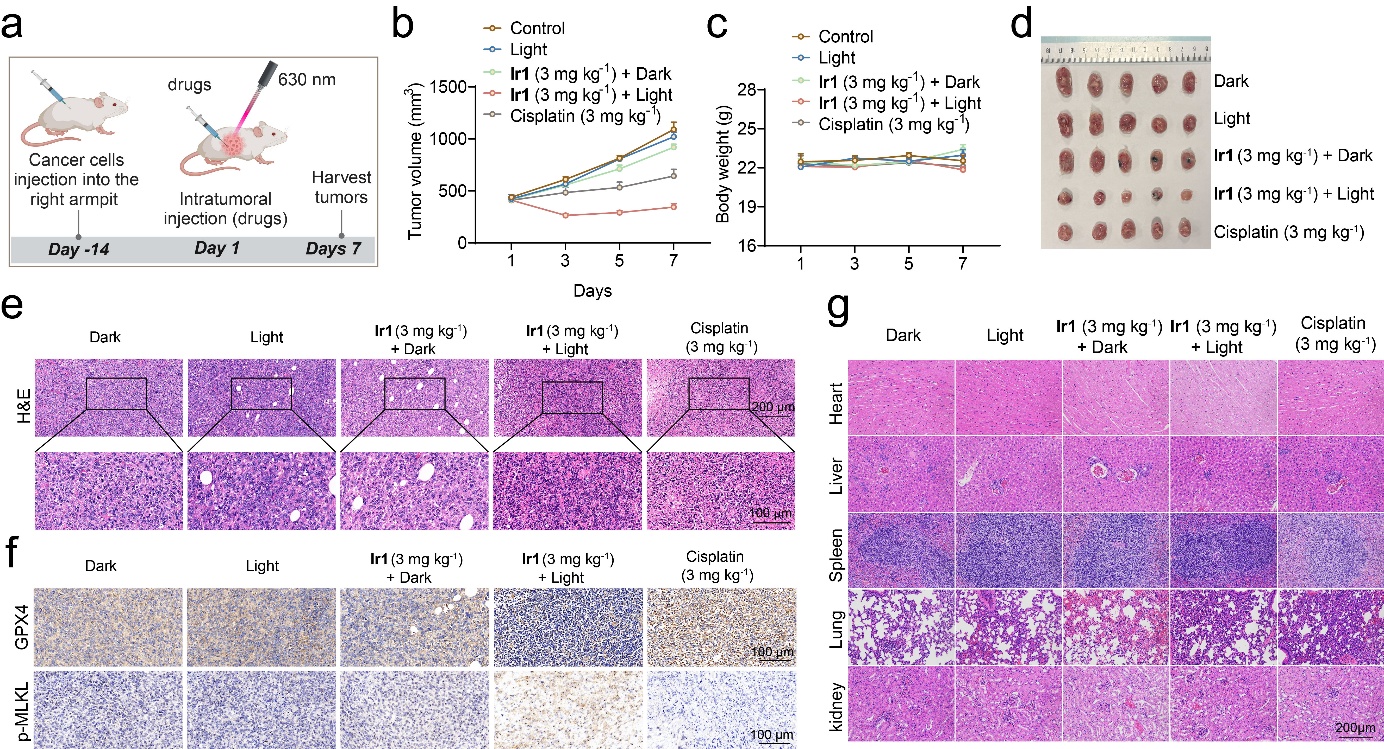


**Figure S29.** (a) Schedule of *in vivo* evaluation. (b) Tumor growth curves over time in different treatment groups (n = 5). (c) Body weight changes in mice during the experimental period (n = 5). (d) Photographic documentation of tumors harvested at endpoint (Day 7, n = 5). (e) H&E sections of tumors. (f) Immunohistochemical staining of tumors. Scale bar: 100 μm. (g) H&E sections of mouse heart, liver, spleen, lung and kidney. Scale bar: 200 μm.


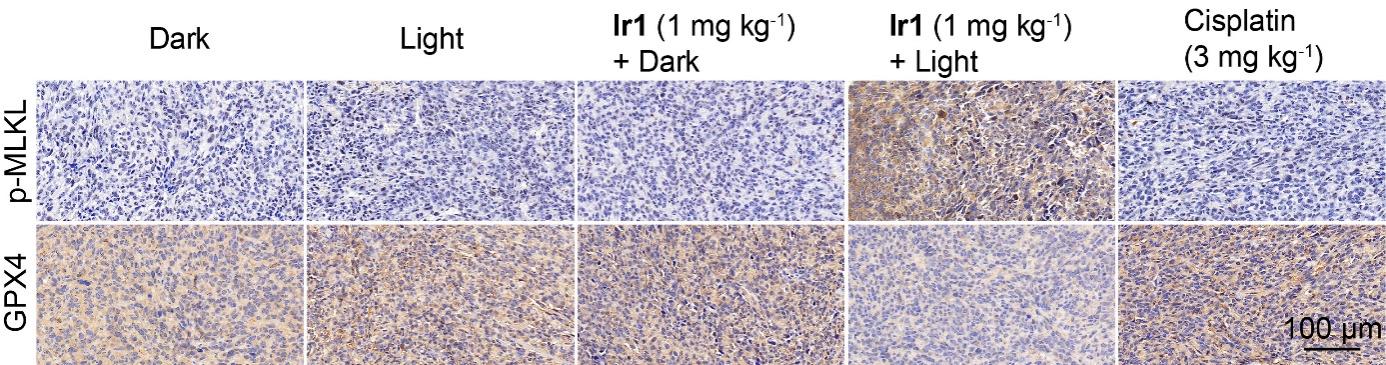


**Figure S30.** Immunohistochemical staining of right-sided tumors. Scale bar: 100 μm.


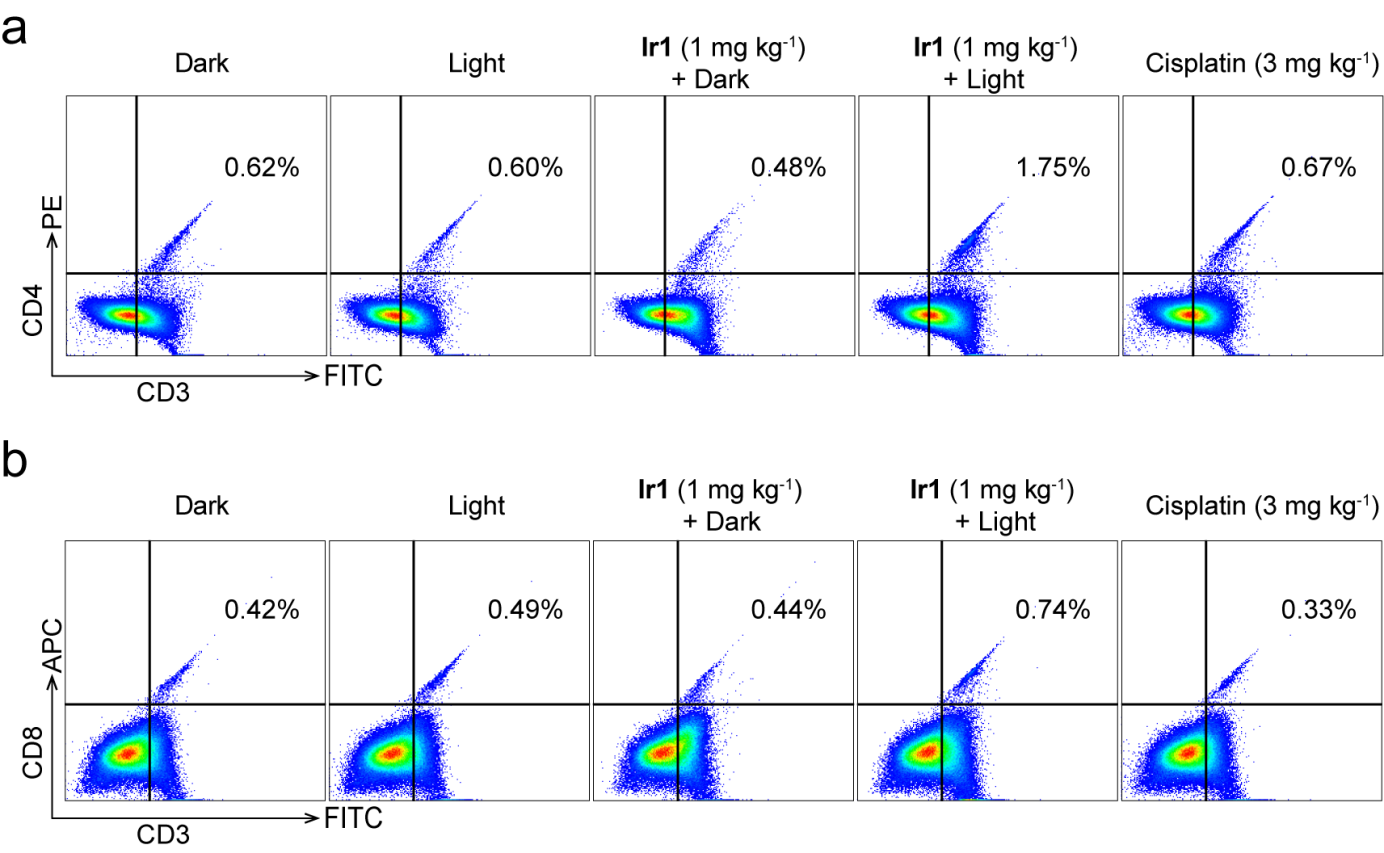


**Figure S31. (a)** Proportion of CD3^+^ CD4^+^ T cells in right-sided tumors. (D) Proportion of CD3^+^ CD8^+^ T cells in right-sided tumors.


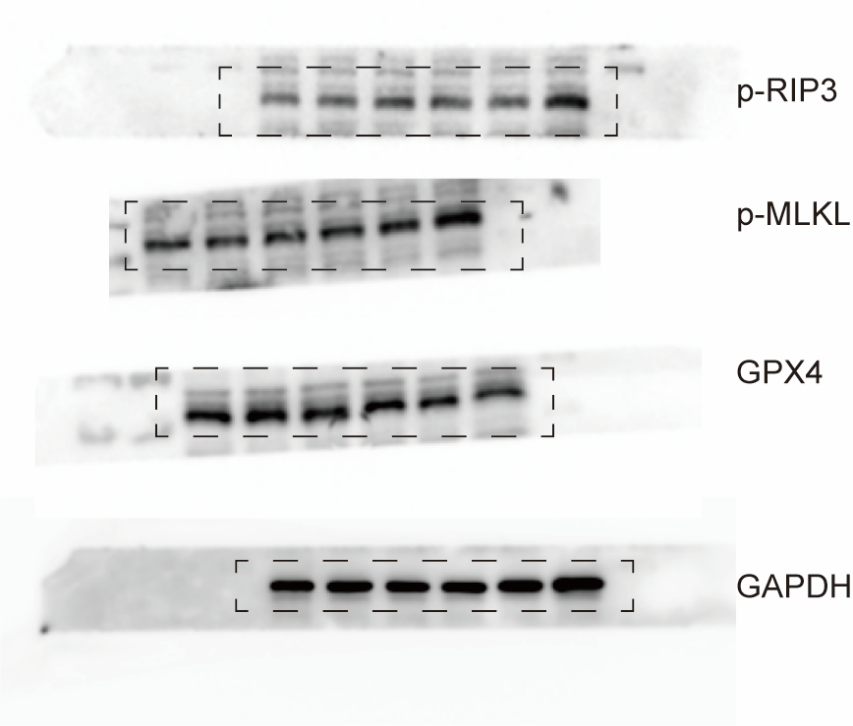


**Figure S32.** Raw images of western blot (Figure 3b).


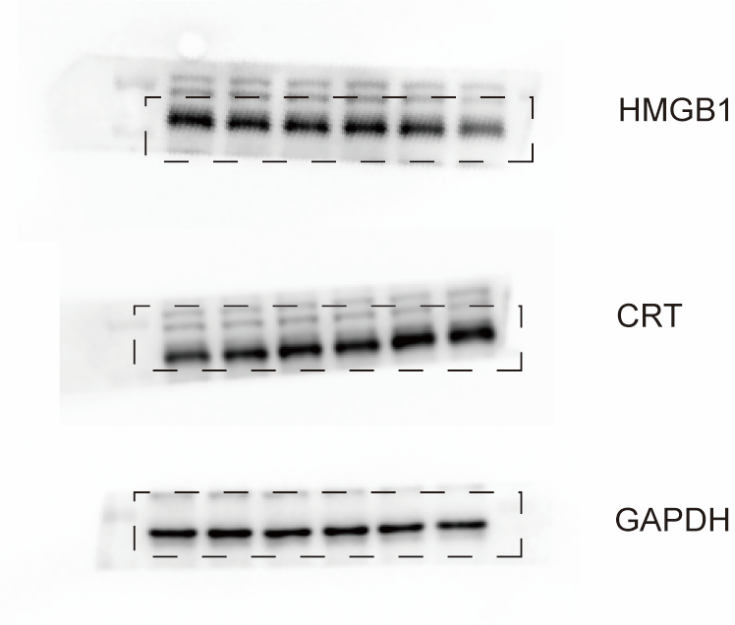


**Figure S33.** Raw images of western blot (Figure 3c).


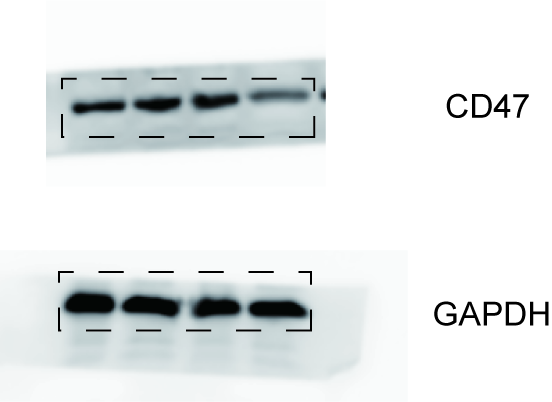


**Figure S34.** Raw images of western blot (Figure 5b).

**Table S1** The excitation energy (eV) values of **Ir1**, **Ir2**, and **L** were collected below.

| Transitions | Excitation Energy (eV) | | |
| --- | --- | --- | --- |
|  | **Ir1** | **Ir2** | **L** |
| S_0_→S_1_ | 2.3904 | 2.4026 | 2.6939 |
| S_0_→S_2_ | 2.5589 | 2.4681 | 3.2419 |
| S_0_→S_3_ | 2.8363 | 2.5741 | 3.7378 |
| S_0_→S_4_ | 2.8784 | 2.5956 | 3.8267 |
| S_0_→S_5_ | 2.9909 | 2.6958 | 4.0370 |
| T_1_→S_0_ | 1.5015 | 1.5166 | 1.6744 |
| T_2_→S_0_ | 2.2111 | 1.8537 | 2.4024 |
| T_3_→S_0_ | 2.2898 | 1.8990 | 2.8551 |
| T_4_→S_0_ | 2.3379 | 2.3185 | 3.0447 |
| T_5_→S_0_ | 2.5250 | 2.4567 | 3.4174 |

**Table S2.** Antiproliferative activity (IC_50_, μM) of **Ir1**, **Ir2** and **L** against breast cancerous (4T1) cell lines.

| Compounds | Normixia | | | | Hypoxia | | | |
| --- | --- | --- | --- | --- | --- | --- | --- | --- |
|  | Dark^[a]^ | Light^[b]^ | | PI^[c]^ | Dark^[a]^ | Light^[b]^ | | PI^[c]^ |
| **Ir1** | 9.97 ± 1.55 | 0.01 ± 0.002 | | 997.00 | 12.81 ± 1.24 | 0.04 ± 0.002 | | 320.25 |
| **Ir2** | ＞ 100 | 56.36 ± 17.4 | | ＞ 1.77 | ＞ 100 | 60.04 ± 6.95 | | ＞ 1.67 |
| **L** | 22.87 ± 6.03 | 16.96 ± 2.60 | | 1.35 | 27.23 ± 1.62 | 12.07 ± 1.15 | | 2.26 |
| Cisplatin | 10.17 ± 0.96 |  |  | | 13.29 ± 0.99 |  |  | |

[a] The cells were incubated with the Compounds for 72 h without irradiation and the cell variability was detected by MTT assay. [b] The cells were incubated with the compounds for 24 h in the dark and irradiated with a 630 nm laser (120 mW cm^-2^, 1 h) and then incubated for 48 h. [c] PI is defined as the ratio of the IC_50_ value in the dark to that obtained in the presence of light.

**Table S3.** Antiproliferative activity (IC_50_, μM) of **Ir1**, **Ir2** and **L** against cervical (HeLa) cell lines.

| Compounds | Normixia | | | | Hypoxia | | | |
| --- | --- | --- | --- | --- | --- | --- | --- | --- |
|  | Dark^[a]^ | Light^[b]^ | | PI^[c]^ | Dark^[a]^ | Light^[b]^ | | PI^[c]^ |
| **Ir1** | 17.85 ± 3.40 | 0.05 ± 0.01 | | 357.00 | 21.79 ± 2.43 | 0.07 ± 0.007 | | 311.29 |
| **Ir2** | ＞ 100 | 12.40 ± 5.46 | | ＞ 8.06 | ＞ 100 | 15.05 ± 3.87 | | ＞ 6.64 |
| **L** | 13.23 ± 1.17 | 8.76 ± 1.29 | | 1.51 | 37.86 ± 4.6 | 13.48 ± 2.15 | | 2.81 |
| Cisplatin | 9.66 ± 0.38 |  |  | |  |  |  | |

[a] The cells were incubated with the Compounds for 72 h without irradiation and the cell variability was detected by MTT assay. [b] The cells were incubated with the compounds for 24 h in the dark and irradiated with a 630 nm laser (120 mW cm^-2^, 1 h) and then incubated for 48 h. [c] PI is defined as the ratio of the IC_50_ value in the dark to that obtained in the presence of light.

**Table S4.** Antiproliferative activity (IC_50_, μM) of **Ir1**, **Ir2** and **L** against colon cancerous (MC38) cell lines.

| Compounds | Normixia | | | | Hypoxia | | | |
| --- | --- | --- | --- | --- | --- | --- | --- | --- |
|  | Dark^[a]^ | Light^[b]^ | | PI^[c]^ | Dark^[a]^ | Light^[b]^ | | PI^[c]^ |
| **Ir1** | 13.30 ± 0.52 | 0.13 ± 0.01 | | 102.31 | 17.07 ± 1.15 | 0.20 ± 0.02 | | 85.35 |
| **Ir2** | 65.06 ± 5.59 | 9.13 ± 2.07 | | 7.16 | ＞100 | 22.47 ± 4.39 | | ＞4.45 |
| **L** | 16.18 ± 0.23 | 11.02 ± 1.83 | | 1.47 | 18.69 ± 0.28 | 13.90 ± 1.07 | | 1.34 |
| Cisplatin | 8.68 ± 0.14 |  |  | |  |  |  | |

[a] The cells were incubated with the Compounds for 72 h without irradiation and the cell variability was detected by MTT assay. [b] The cells were incubated with the compounds for 24 h in the dark and irradiated with a 630 nm laser (120 mW cm^-2^, 1 h) and then incubated for 48 h. [c] PI is defined as the ratio of the IC_50_ value in the dark to that obtained in the presence of light.
